# Modeling Complex Developmental Disease: The Case of Polycystic Kidney Disease

**DOI:** 10.3390/jdb14030036

**Published:** 2026-08-10

**Authors:** Jay DeLoriea, Cody Casey, Lexee Shearer, Victoria Chen, Angelica Bryant, Chiara Gamberi

**Affiliations:** 1 Biology Department, Coastal Carolina University, Conway, SC 29526, USA; jmdelorie@coastal.edu (J.D.); cmcasey@coastal.edu (C.C.); ljshearer@coastal.edu (L.S.); victoriaacchenn@gmail.com (V.C.); asbryant1@coastal.edu (A.B.); 2 Carolina Forest High School, Myrtle Beach, SC 29579, USA; 3 Disease Modeling Research Collaborative, Coastal Carolina University, Conway, SC 29526, USA; 4 Hetzer Center for Biomedical Sciences, Coastal Carolina University, Conway, SC 29526, USA

**Keywords:** polycystic kidney disease, development, genetic models, genetic networks, *Drosophila*, disease models, drug discovery

## Abstract

Both genetics and the environment affect the phenotypes of polycystic kidney diseases (PKD), such as autosomal dominant (AD) PKD, autosomal recessive (AR) PKD and nephronophthisis (NPH). Variable phenotypes, pleiotropy, divergent severity and progression, even in family members who inherited the same disease-causing mutation(s), signal the involvement of networked genes and modifiers. Several PKD-linked genes function in development and renal tubule morphogenesis. Cystic renal tissues feature metabolic remodeling, functional reprogramming and dysregulation of several shared factors and pathways. ADPKD, ARPKD and NPH partially phenocopy each other. Understanding the developmental arc of cystic kidney disease and its complex phenotypes would improve diagnostics and help develop effective personalized treatments. However, this is challenging to study in vertebrate systems due to genetic redundancy, functional overlap, and a dearth of genetic tools. Underused in this context, *Drosophila melanogaster* offers high genomic and pathway conservation, a wealth of genetic tools, and rapid generation times, making it a reliable and sustainable model for mechanistic, genome-wide, and precision medicine studies. Here, we surveyed ADPKD, ARPKD, and NPH, compared renal and extrarenal phenotypes, and examined the network of shared and unique contributors, their healthy and diseased functions and conservation from the perspective of mechanistic whole-animal modeling.

## 1. Introduction

Polycystic kidney diseases (PKD) display more than one causative gene and, typically, variable manifestations due to a combination of genetics and the environment. Mutations in either the human *PKD1* (80–85% of the cases) or *PKD2* (10–12% of the cases) genes account for the majority of autosomal dominant (AD) PKD cases [1,2,3], with the remaining instances (7–8%) being linked to mutations in other genes [4,5]. *POLYCYSTIC KIDNEY AND HEPATIC DISEASE 1* (*PKHD1*) mutations account for the majority of autosomal recessive (AR) PKD cases [6,7,8,9]. Mutations in several *NPHP* genes are implicated in nephronophthisis (NPH), also a cystic kidney disease [10]. The cystic phenotypes are highly variable clinically depending on the inherited mutation and largely unknown variants elsewhere in the genome that modify disease presentation and progression [11,12]. The complexity of cystic kidney disease is thought to result from the dysregulation of several developmental networks important for renal tubule morphogenesis and metabolism that predispose to cyst formation and unfold their effects over time and in context-specific ways. The polygenic nature of PKD and the disruption of multiple cellular networks underlie the typical phenotypic variation and complicate prognosis and treatment [11,13]. Here, we will systematically examine the cystic phenotypes, the shared and unique factors implicated in ADPKD, ARPKD and NPH, their developmental function in healthy and diseased renal tubules, and their conservation from the perspective of disease modeling using traditional rodent and non-traditional (in PKD) whole-animal developmental models like *Drosophila*.

## 2. ADPKD

Likely underdiagnosed, ADPKD (OMIM 173900) is estimated to affect 1 in every 400 to 1000 people worldwide based on genetic data and 500,000 people in the United States alone [14,15,16,17,18]. The major *PKD1* and *PKD2* genes encode, respectively, the polycystin 1 (PC1) and 2 (PC2) transmembrane proteins [1,2,3]. In mice, the corresponding orthologs are called *Pkd1* and *Pkd2*, and the cognate proteins are abbreviated as Pc1 and Pc2. Typically, ADPKD becomes symptomatic in late adulthood, due to the accumulation of fluid-filled cysts in the renal tubules, or nephrons [1,2]. As cysts grow, they increase pressure on the surrounding tissues, obstruct nearby nephrons and vasculature, and eventually lead to kidney failure [19]. In early (E) and very-early-onset (VEO) cases, the phenotype becomes evident already in utero or shortly after birth, and diagnosis is immediate [20,21,22]. ADPKD is progressive and potentially lethal, being a leading cause of renal replacement therapy (RRT) [23]. A 19-year-long study found that 20,483 of 314,176 RRT recipients from 12 different countries had ADPKD and outnumbered all other conditions [24]. Besides *PKD1* and *PKD2*, several genes have also been found mutated in PKD patients (discussed below).

**Table 1:** (**A**) Renal-associated manifestations and incidence of ADPKD, ranked by incidence. (**B**) Extrarenal manifestations and incidences of ADPKD, ranked by incidence.

Symptom	Frequency
(**A, renal**)	
Fluid-filled cysts	100% (*n* = 1000 bootstrap samples) [25,26]
Enlarged kidneys	100% (*n* = 242 PKD patients) [27]
End-stage renal disease/end-stage kidney disease (ESRD/ESKD)	75% by 70 years old, (*n* = 741 patients with ADPKD) [2,28]
Abdominal fullness/pain	61.4% (*n* = 171; 94 women, 77 men) [29]
Defective urine concentration	≥60% in children (*n* = 10) [2,30,31]
Cyst hemorrhage and/or gross hematuria	≤60% of adult patients, (*n* = 191) adult ADPKD subjects [2,32,33]
Hematuria	35–50% (*n* = 191 adult ADPKD subjects) [32,34,35]
Urinary tract infections (UTI)	30–50% (*n* = 5 women and 2 men, 29 to 65 years old) [2,33,36]
Nephrolithiasis	20–25% of ADPKD patients (*n* = 74) [37,38]
Proteinuria	14–34% (*n* = 270 ADPKD patients) [39]
Kidney cyst infection	7.5% (*n* = 389 ADPKD patients) [40]
Renal cell carcinoma (RCC)	<1% of adult patients (*n* = 96 RCC cases) [2,41]
(**B, extrarenal**)	
Arterial hypertension	86.6% (*n* = 411) [42]
Diverticulosis	75% (*n* = 18) [43]
Hypertension	50–70% prior to eGFR ^1^ decline, 20–40% in children (*n* = 284) [2,44,45,46]
Hepatic fibrosis	64% (*n* = 1230) [47]
Polycystic liver disease (PLD)	58% ages 15–24, 85% ages 25–34, and 94% ages 35–46 years (*n* = 230 ADPKD patients) [48]. >80% of patients by 30 years old (*n* = 434) [2,49]
Seminal vesicle cysts	60% (*n* = 45, males) [50,51]
Abdominal wall hernias	45% (*n* = 85) [34,52]
Fluid-filled pancreatic cysts	36% (*n* = 110 ADPKD patients) [53]
Pericardial effusion	35% of adult ADPKD patients (*n* = 60) [2,54]
Bronchiectasis	≤35–40% of adult patients [2,33]
Male infertility	28.5% (*n* = 28 males) [55]
Aortic root dilatation	≤25% (*n* = 228) [2,45]
Mitral valve prolapse or bicuspid aortic valve	≤25% of adult patients (*n* = 163 patients with ADPKD) [2,45].
Heart valve disease	14.4% (*n* = 397) [34,42]
Intracranial aneurysm	10% (*n* = 266) [56]
Arachnoid cysts	8% of adult patients [2,33]

^1^ eGFR (estimated glomerular filtration rate).

### 2.1. ADPKD Phenotypes, Genetics and Pleiotropy

**Figure 1:**
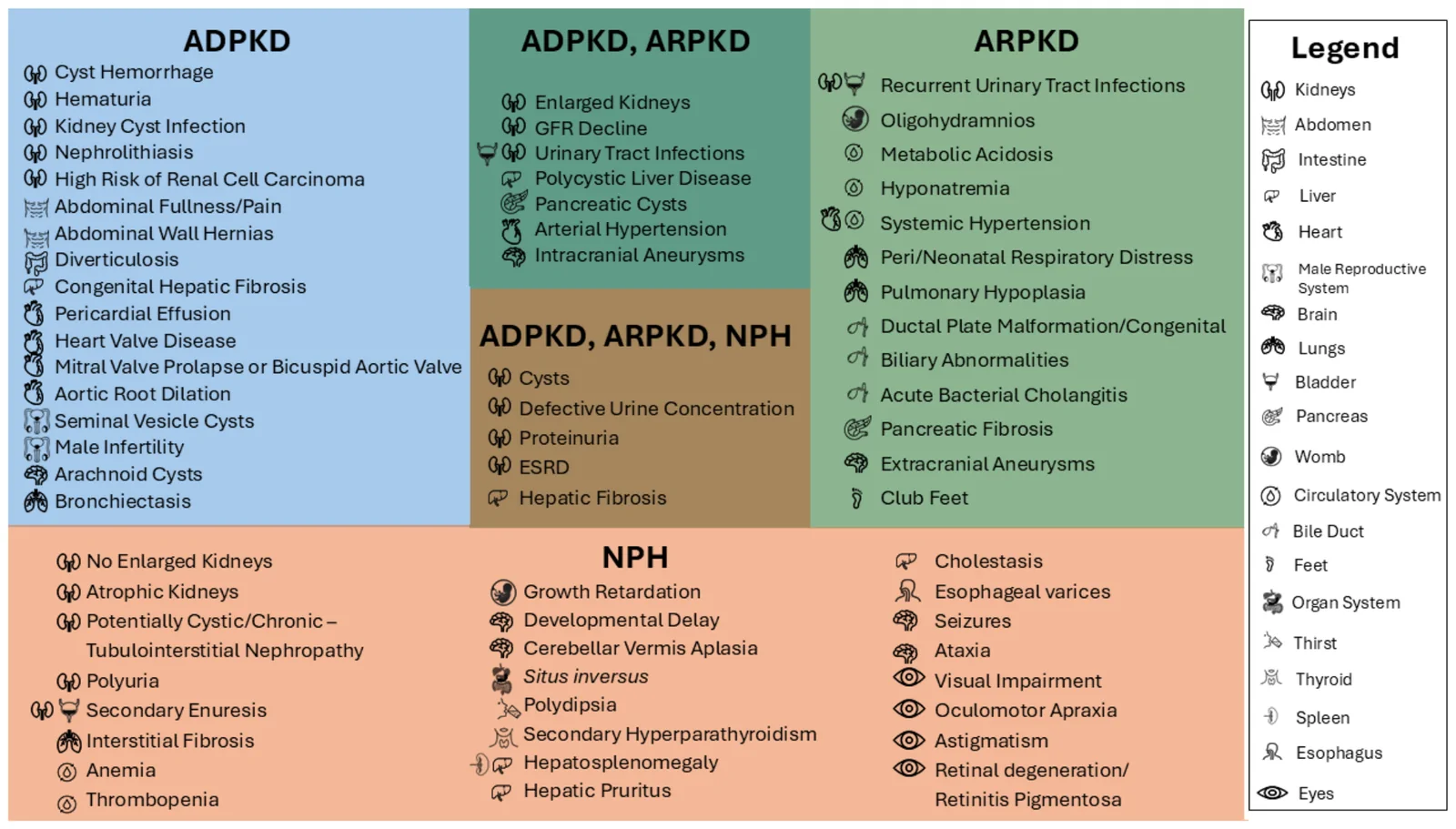
**Renal and extrarenal clinical manifestations of ADPKD, ARPKD and NPH**. Unique and shared phenotypes are indicated, grouped by organ involvement. See text for details. GFR (glomerular filtration rate), ESRD (end-stage renal disease).

Typical renal manifestations in ADPKD include numerous cysts forming along the entire renal tubule, but most frequently in the terminal region (Table 1A, Figure 1) [1,2]. The ADPKD cysts develop as expansions of the renal tubular epithelium, either in continuity with the tubule itself, or separated from it as external “bubbles”, with a round or fusiform shape [2,57]. Additionally, patients also display hematuria, urinary tract infections, and nephrolithiasis, accompanied by (non-renal) early-onset hypertension, abdominal fullness and pain (Table 1, Figure 1) [2,3,33,45]. Additional variable extrarenal manifestations include polycystic liver disease (PLD) in 80% of cases, pericardial effusion, male infertility and cysts in the arachnoid, seminal vesicle, liver and pancreas, permanently widened airways (bronchiectasis), intracranial aneurysms, diverticulosis and abdominal hernias, and cardiovascular abnormalities (Table 1B) [33,45,49,51,55,58,59,60,61,62,63,64]. The complex phenotypic presentations of ADPKD are consistent with pleiotropic effects and with the wide expression pattern of the polycystins. Typically, ADPKD also displays inflammation and fibrosis [33,65,66]. The genetics of ADPKD is complicated. More than 2500 different *PKD1* and *PKD2* mutated alleles have been described, each affecting fewer than 2% of the total patient population in the ADPKD Mutation Database [67,68]. Moreover, the human genome may contain at least six *PKD1* pseudogenes with up to 99% sequence homology [67]. Due to their high homology, these pseudogenes may confuse sequencing efforts to determine specific mutations. Human *PKD1* and *PKD2* and murine *Pkd1* and *Pkd2* homozygosity are usually embryonic lethal; however, somatic loss of heterozygosity in the cells of the renal tubule is thought to fuel PKD-type cyst development, according to the “two-hit” model inspired by the oncology field [69,70,71]. Patients with biallelic mutations described as compound heterozygous or homozygous pairs with one weak copy of *PKD1* or *PKD2* may survive [28]. Alternatively, the threshold model posits that a polycystin activity drop below 20% may trigger cyst formation [72,73,74]. About 2% of patients with severe ADPKD (resembling ARPKD) and carrying two incompletely penetrant *PKD1* alleles in trans were found to harbor additional mutations in PKD-related genes, e.g., *HNF-1B* [75,76]. In genetically unresolved (GUR) ADPKD, patients are diagnosed with PKD, but no mutations in *PKD1* or *PKD2* are detected. In these cases, whole-exome sequencing may be used in search of causative mutations in other genes. GUR PKD presents itself at different severities within the spectrum typical of *PKD1*-dependent ADPKD. One family with GUR-ADPKD with mild kidney and strong liver cystic disease was found to segregate a missense variant in *GANAB*, encoding an endoplasmic reticulum glycosylase [77]. Analysis of 321 additional GUR ADPKD families identified eight more *GANAB* mutations: three frameshift, two splicing, two missense, and one nonsense mutations [77]. Mutations in several other genes produced PKD-like phenotypes, e.g., *intraflagellar transport protein 140* (*IFT140*, see ciliary Section 2.2 and Section 2.4 below), *NIMA-related kinase 1* (*NEK*), *DnaJ Heat Shock Protein Family* (*Hsp40*) *Member B11* (*DNAJB11*), [64] and oral–facial–digital syndrome type 1 (*OFD1*). The *OFD1* gene is located on the X chromosome and is often male–lethal prenatally; thus, mutations largely affect women [78]. *OFD1* mutations produce renal cysts in 50% of cases with hepatic and pancreatic cysts, as well as typical cleft palate, digital deformity and cognitive impairment, usually in adulthood [79,80,81,82]. One early-onset PKD case associated with an *OFD1* mutation was reported recently [83]. Thus, *PKD1* and *PKD2* loss-of-function are most frequently found in ADPKD patients, and mutations in several other genes can yield similar phenotypes.

### 2.2. Cilia in Development and PKD

A portion of the cellular pool of polycystins, fibrocystin (mutated in ARPKD) and nephrocystin-1 (implicated in nephronophthisis, NPH, discussed below) are found in or adjacent to the primary cilium. Thus, ADPKD, ARPKD, and NPH all seem to affect the primary cilium and are often considered ciliopathies displaying varying proportions of cysts, inflammation, and fibrosis [3,84,85,86,87,88]. Cilia are conserved microtubule-based organelles of developmental importance that protrude from the apical surface of most cell types [84,87,89]. Usually several for each cell, motile cilia contain nine peripheral and one central microtubule pair conferring motility. Motile cilia contribute to the nodal flow establishing visceral left–right asymmetry during vertebrate embryogenesis and coordinate cell proliferation, differentiation, and signal transduction pre- and post-developmentally [87]. In contrast, non-motile primary cilia are found one per cell, lack the central microtubules for motility, and are thought to sense external stimuli through chemo-, photo- and mechano- sensors [84,90,91,92]. In the mammalian embryo, primary cilia detect nodal fluid flow to establish right–left symmetry [93]. The primary cilia originate from a centriole-derived basal body located next to the cell membrane that nucleates microtubule outward growth [92]. Collectively, the microtubules form the central axoneme, delimited by a specialized membrane in continuity with the cell membrane that is enriched in receptor and channel proteins; at the base, a transition zone gates materials entering the cilium. A complex intraflagellar transport (IFT) system moves cargo from the ciliary base distally using kinesin-2 and proximally using the dynein-2 motor proteins [92]. Several conserved receptors are implicated in signal transduction cascades and are also found at the cilium, notably, Hedgehog, transforming growth factor beta (TGF-β) and G-protein coupled receptors (GPCR) [94,95,96]. Other signal transduction pathways regulated by cilia include Wnt-dependent Planar Cell Polarity (PCP) and several receptor protein kinases [84,97]. Similarly, in the renal tubule, primary cilia are thought to be sensing fluid flow and pressure and capturing long-range chemical signals, e.g., morphogens and growth factors normally activating intracellular transduction cascades that are typically disrupted in PKD [98] and are further discussed in Section 2.4.

### 2.3. Factors Implicated in ADPKD

**Figure 2:**
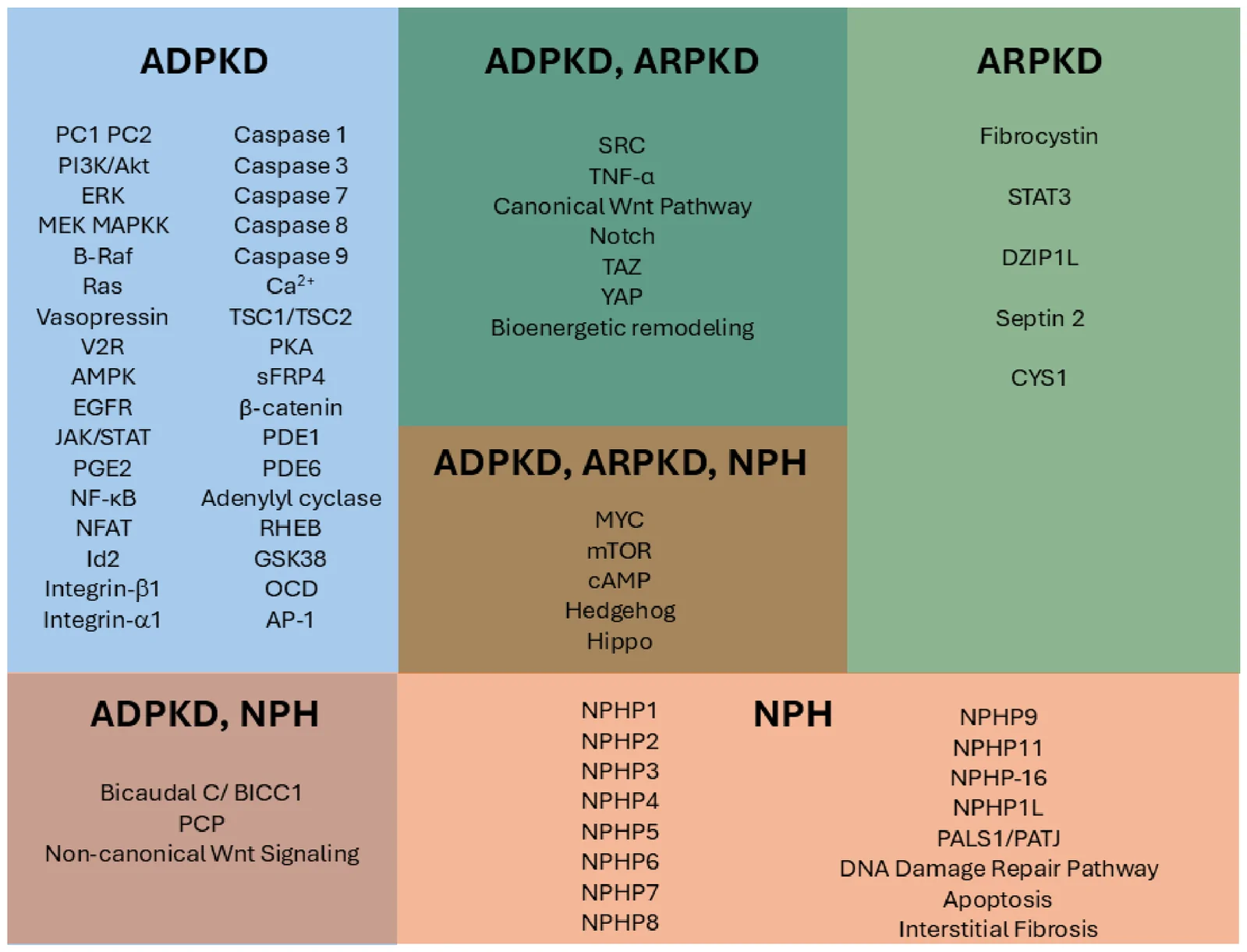
**Factors implicated in ADPKD, ARPKD, and NPH.** Unique and common factors are indicated. See text for details.

Numerous factors contribute to ADPKD cysts. Polycystin loss-of-function disrupts several regulators of cell growth, polarity, metabolism, and death that are conserved evolutionarily. In ADPKD, these factors together remodel the renal tubule cells metabolically and morphologically, similar to neoplasia [99]. For some of these factors, the dysregulation per se is sufficient to cause cysts, as first described for murine c-Myc [100]. However, frequently the resulting phenotype does not exactly coincide with PKD. Even after accounting for the properties of different mutations, these results suggest that many of these factors contribute to the cystic phenotype without being necessary or sufficient (also see Section 2.4 below). Thus, what precisely defines PKD-like cyst remains undefined and may be encoded both in the factors’ identities and activities, and in the relationships between such factors, i.e., their network properties. Below, and in Table 2, we summarize the functions of several factors implicated in ADPKD and examine their conservation in *Drosophila*, an established genetic model in which causative and network studies are feasible (Table 2, Figure 2).

#### 2.3.1. PC1 and PC2

Directly linked to ADPKD, (human) PC1 and PC2 are transmembrane proteins found throughout the cell and in the primary cilium of the tubular epithelial cells. PC1 is an orphan GPCR [101,102], while PC2 is a conserved member of the transient receptor potential (TRP) family of ion channels that transports cations and indirectly affects intracellular Ca^2+^ [103,104]. Functional PC1 and PC2 interact through their C-terminal coiled-coil motifs to form heterodimeric complexes [105]. Compared to the well-described TRP receptor family, the function(s) of both PC1 and the PC1-PC2 complexes are poorly understood. Studies in ADPKD models showed that the polycystins prevent cyst formation by inhibiting the cilia-dependent cyst activation (CDCA) signaling, and PC loss-of-function yields cyst formation and luminal expansion of the renal tubule. *Pkd1* and *Pkd2* mutant mice (without ciliary Pcs) displayed cystic growth, and Pcs re-expression reversed PKD phenotypes presumably by suppressing cyst formation [106]. PCs also function as regulators of lumen contraction depending on the relationship between PC1/PC2 and the CDCA in the cilia [107]. Unlike the *PKD1* gene, *PKD2* has a *Drosophila* homolog, *Almost there* (*Amo* [104], discussed below).

#### 2.3.2. Myc

Upregulated in both dominant and recessive PKD, Myc belongs to a gene family of developmentally important transcription factors tightly regulated both transcriptionally and post-transcriptionally that are activated downstream of several pro-proliferative signaling cascades altered in PKD, including Wnt [108], Sonic Hedgehog-Gli [109], Hippo [110], Notch [111], and EGF [112,113]. Myc activation promotes cell proliferation and the associated metabolic upregulation to sustain the needs of dividing cells in normal development and neoplastic pathologies [114]. In the ADPKD renal epithelium, Myc upregulation and cell growth co-exist with caspase-3-mediated cell death, with the former possibly more important at cyst initiation and the latter more preponderant later during cystic growth [115,116]. Myc is conserved in *Drosophila*, and the human and fly proteins are functionally interchangeable [117,118,119].

#### 2.3.3. mTOR

The mammalian target of rapamycin (mTOR) signaling is activated at least downstream of PC1 activation by the primary cilia signal. Normally, PC1 and tuberin inhibit mTOR activity; when defective, persistent cell de-differentiation and proliferation, interstitial fibrosis and cyst growth occur [120]. Immunohistochemistry of kidney tissue from advanced-stage ADPKD with antibodies against phosphorylated (phospho-) mTOR and phospho-S6 kinase (early mTOR target) revealed both elevated mTOR activity and high levels of phospho-S6 kinase in the cyst-lining epithelial cells and the early cystic lesions of 5-week-old *Pkd1^−/−^* mice [121]. The late-onset PKD model Oak Ridge polycystic kidney (Orpk) rescue mice carried both *Ift88/Tg727* mutations and an *Ift88/Tg737Rsq* transgene [122]. When treated with the mTOR inhibitor rapamycin, the cystic index was reduced, indicating that mTOR activation contributes to cyst formation [121]. Similar results have been found in other PKD models [123,124,125,126,127], including *Drosophila* [128]. Recent findings in a *Pellino1* (*Peli1*) transgenic mouse also confirmed a role for activation of mTOR/S6K signaling in renal cyst formation [129].

#### 2.3.4. c-Src

A ubiquitous proto-oncogenic membrane-associated non-receptor tyrosine kinase and signal transduction intermediary, c-Src mediates cell-surface signaling, adhesion, and growth [130]. In fibroblasts, Src was found in endosomes, secretory vesicles, and the inner leaflet of the plasma membrane [131]. In tubular epithelial cells, c-Src was anchored to the basolateral membrane via lipidic residues, and its activation triggered PI3K/AKT signaling and synthesis of cell cycle regulators [132]. Both c-Src levels and activity were increased in human ADPKD epithelial cells and in the kidneys of *C57/Pkd1* heterozygous mice [133]. Consistent with a role in PKD-type cyst pathology, SKI-606/bosutinib, a Src and Abl inhibitor used in oncology, reduced the higher-than-normal cell–matrix adhesion of ADPKD cultured cells [133]. In vivo, SKI-606 significantly reduced cyst numbers and normalized kidney volume, weight, and morphology in comparison to untreated (or vehicle-treated) *C57/Pkd1* mice [133].

#### 2.3.5. Vasopressin and Cyclic AMP

Arginine vasopressin (AVP, also known as anti-diuretic hormone, ADH) concentrates urine in response to elevated plasma osmolality to maintain fluid homeostasis. Physiologically, vasopressin is secreted from the pituitary gland and binds to the V2 receptor (R) in the collecting ducts, which triggers apical redistribution of the aquaporin-2 transmembrane channel in the tubular cells. Water is then reabsorbed and dilutes the circulating fluid [134]. In early ADPKD, patients become dehydrated, which triggers vasopressin-V2R signaling, increases levels of intracellular 3′-5′-cyclic adenosine monophosphate (cAMP), and promotes cystic cell proliferation [135]. Consistent with this model, in a water deprivation test, early ADPKD patients displayed compromised urine concentration capacity [136]. Over time, dehydration taxes the cardiovascular system and contributes to the typical PKD complications [137,138,139]. ADPKD (and ARPKD, below) cells have an increased vasopressin-specific cAMP response thought to stem from cystic cells derived from collecting ducts [140]. Because vasopressin stimulates cAMP production in collecting ducts and the distal portion of the nephrons, the predominant cyst formation sites in PKD, these observations first indicated that cAMP contributed to all stages of PKD [141]. Consistent with clinical observations, several PKD models including *pcy*, *jck*, and *Pkd2^-/tm1Som^* mice and *PCK* rats confirmed a pattern of elevated renal cAMP levels [137,142,143,144]. Molecularly, cAMP regulates cell proliferation, differentiation, transcription, electrolyte and fluid transport. cAMP levels reflect the balance between adenylyl cyclases catalyzing cAMP formation from ATP and phosphodiesterases degrading cAMP into AMP [137]. Elevated cAMP levels in both AD and ARPKD activated the mitogen-activated protein kinase kinase (MAPKK)/extracellular regulated kinase pathway (MEK/ERK) and promoted cystic growth and secretion [140,145,146]. Inhibition of the vasopressin-V2R pathways through the V2R agonist tolvaptan (discussed below) has been shown to lessen cystic growth and slow disease progression [147].

#### 2.3.6. cAMP, Raf/MEK/ERK Signaling Pathway

Dysregulation of intracellular Ca^2+^ starts the cAMP cascade by reactivating Akt, which relieves B-Raf inhibition and elevates cAMP. The latter activates ERK, B-Raf and cell proliferation and survival, and coordinates cell-cycle re-entry during tissue repair [145,148,149]. In PKD, receptor tyrosine kinases (RTKs) bound to growth factors trigger phosphorylation cascades that activate small G-protein RAS, followed by ERK, MEK, and RAF, MAPKK and the extracellular signal-regulated kinase ERK1/2, which phosphorylate modulators of growth, proliferation, differentiation, migration and survival [150]. In both ADPKD and ARPKD, MEK/ERK activation becomes a pro-proliferative signal for the cystic epithelial cells, yet seemingly inhibits cell proliferation in normal human kidneys [135,151].

#### 2.3.7. Tumor Necrosis Factor Alpha (TNF-α)

TNF-α and other pro-inflammatory cytokines were initially found in the kidney and cystic fluids of individuals with ADPKD undergoing therapeutic cyst decompression, nephrectomy or biopsy [152,153]. A study of 233 participants included in the German AD(H)PKD database (The German ADPKD Tolvaptan Treatment Registry (NCT02497521) found increased TNF-α in patients compared to healthy controls and a negative correlation between TNF-α levels and eGFR (estimated glomerular filtration rate, a measure of kidney function), suggesting that increased TNF-α contributes to cyst growth and ADPKD progression [154]. The renal epithelial cells of carriers of *PKD1* and *PKD2* mutations exhibited a higher nuclear fraction of inhibitor of DNA binding/differentiation 2 (Id2), contributing to abnormal epithelial cell proliferation and differentiation in the kidneys [155]. The receptor activator of the NF-κB ligand (RANKL) protein increased the TNF-α mRNA in both wildtype and *Pkd1^−/−^* mutant mouse embryonic kidney (MEK) cells via NF-κB signaling and amplified Id2 protein expression post-transcriptionally [154]. Notably, mTOR activity is needed for Id2 upregulation because rapamycin inhibition completely blocked Id2 production in *Pkd1* null mouse embryonic kidney cells [154]. Thus, mTOR overactivation reinforces TNF-α production in PKD. TNF-α was also found to promote infiltration of pro-inflammatory macrophages that contribute to cyst growth [154]. The cyst-lining cells are thought to be fueled by a cancer-like TNF-α-dependent loop. In *Pkd1* loss-of-function renal epithelial cells, the TNF-α receptor TNFR1 was upregulated [156]. Moreover, during cyst development, TNF-α was secreted in the cyst fluid, and the TNF-α–TNFR1 interaction was stabilized, which fueled a positive TNF-α autoregulatory loop and made the cyst-lining cells more dependent on TNF-α signaling than the adjacent non-cystic cells [157]. Such a differential could be exploited pharmacologically by repurposing mimics of the second mitochondria-derived activator of caspases (Smac) used in oncology to preferentially activate apoptosis in the TNF-α-dependent cystic cells. Consistent with this scenario, one such molecule, the birinapant analog GT13072 [157], and three Smac mimics of novel synthesis [158] exhibited cyst-reducing activity, respectively, in a murine and a Drosophila PKD model.

#### 2.3.8. Wnt/WNT

WNT signaling is critically involved in tissue morphogenesis and in establishing PCP via a canonical WNT/β-catenin branch and a β-catenin-independent pathway involving the Rho and Rac GTPases [53,159,160,161]. In the canonical WNT pathway, when WNT binds to receptors of the Frizzled family, β-catenin is stabilized, moves to the nucleus, and interacts with the T-cell factor (TCF)/Lef1 transcription factors to induce target gene expression [53]. β-catenin displaces the transcriptional repressor Groucho within the TCF/Lef effector complex and recruits coactivators to regulate the expression of target genes *c-MYC* and other WNT pathway components like *AXIN2* [161]. The cytoplasmic tail of PC1 (PC2-interacting domain) affects WNT signaling [108]. Over-expression of the PC1 C-terminal domain in human embryonic kidney (HEK293T) cells stabilized β-catenin and its downstream signaling [108]. Similarly, expressing a stable β-catenin mimicked these effects [162]. Non-canonical Wnt signaling and PCP (connected to ciliary dysfunction, see below) are also dysregulated in PKD tissue [161,163,164]. In renal tubular cells, PCP is necessary developmentally to form tubules with regular diameter and is normally attenuated in the adult; however, in PKD models, PCP protein distributions remain unaffected [164]. Interestingly, PCP dysregulation was not sufficient to induce cysts without an additional proliferation signal [164]. Moreover, the PCP and ciliary contribution to cysts appeared to be independent of each other [164]. *Pkd2* mutation increased β-catenin signaling and cystogenesis in mouse embryonic fibroblasts, collecting tubule cells and kidney renal epithelia [165]. In a different system, PC1 over-expression in embryonic kidney cells only activated activator protein 1 (AP-1), which underscores the complexity of the networks implicated in renal cyst formation [166]. Consistent with a role in PKD, genetic and pharmacological Wnt downregulation reduced the cystic index [165]. Aberrant activation of canonical Wnt signaling was found in several *PKD1* and *PKD2* loss-of-function systems [163,167,168,169]. Moreover, WNT/β-catenin, c-MYC, and TAZ are normally functionally linked, and their relationship is disrupted in PKD (see below). Possibly relevant for ADPKD, two mutations in the human *BICAUDAL C (BICC1)* gene were found to be associated with an over-active WNT pathway in individuals with renal cystic dysplasia [170]. Suggesting functional conservation, the Bicaudal C (BicC) regulation of Wnt pathway components was first observed in the *Drosophila* ovary [171].

#### 2.3.9. Hippo

Implicating the Hippo pathway as a mediator of PKD-type cystogenesis, tissue from individuals with AD (and AR) PKD with renal cystic tumors showed altered Hippo signaling [172]. Transcriptomics analysis of murine *Pkd1^−/−^* cells also revealed Hippo pathway dysregulation [173]. Higher-than-normal nuclear localization of YAP, the end product of the Hippo pathway, in cystic compared to pre-cystic tubules supported the notion that Hippo may be dysregulated and supportive of cyst growth yet not causative for cystogenesis [174]. The Hippo downstream effector YAP repaired cell damage in the early stages of PKD in several models [172,175], but it accumulated in the nucleus, promoting pathological transcriptional activation in late-stage PKD [172]. Downstream Hippo effector TAZ normally interacts with PC1 to inactivate β-catenin. In *PKD1^−/−^* (human) null renal tubule cells, TAZ interacted instead with AXIN1 and accumulates in the nucleus together with β-catenin, which activates pro-proliferative c-MYC [176]. Thus, the TAZ/Wnt/β-catenin/c-MYC axis is thought to affect PKD-type cystogenesis. Human *PKD1^−/−^* () and murine *Pkd1^−/−^* cells also displayed nuclear accumulation of YAP and TAZ and transcriptional activation of their respective targets, including *c-MYC* [173], implicating the RhoA/YAP/c-MYC axis in PKD [110,173]. Independent of its role in cell proliferation and apoptosis, YAP functions downstream of Cdc42 to regulate cell fate and tubular morphogenesis in the developing nephron [177].

#### 2.3.10. Integrin-β1 and Integrin α-1

Part of a protein family with eight human members, the integrin-β and α subunits form heterodimers that connect to the extracellular matrix and the cytoskeleton [178]. In so doing, integrins transform mechanical stimuli into cellular responses. Integrin-β1 is enriched in the kidney and colocalizes with PC at the focal and cell–cell adhesions, as well as the cilia [179,180,181,182]. Collecting duct-specific conditional double knockout mice *Pkd1^−/−^ Itgb1^−/−^* were protected from cyst formation, cyst progression, and fibrosis [183]. These observations placed integrin-β1 downstream of *Pkd1* and made it a likely *Pkd1* modifier. A similar rescue was found in *Ift88^−/−^ Itgb1^−/−^* mice in which the cystic phenotype is linked to loss of cilia [184]. A qualitatively similar rescue was also observed in “global” double knockouts *Pkd1^nl/nl^ Itga1^−/−^* that exhibited smaller cysts, reduced interstitial fibrosis and less tubular atrophy [185]. Thus, integrin complexes in the renal tubular cells appear to mediate cyst formation and progression in ADPKD.

#### 2.3.11. PI3k/Akt

PI3K, Akt, and mTOR kinases regulate cellular proliferation. Activation of the PI3K/Akt/mTOR pathway is triggered by growth factor receptor binding [186,187]. Class I PI3Ks produce the phosphatidyl inositols Ptdlns-3,4,5-P3 that recruit, among others, members of the AKT sub-family of AGC serine/threonine kinases. AKT phosphorylation is an indicator of class 1 PI3K activation [188,189]. AKT phosphorylates tuberous sclerosis complex (TSC), which inhibits Ras Homolog Enriched in Brain (RHEB) and releases mTOR from the mTOR Complex 1 (mTORC1). When mTOR is released/activated, multiple cellular growth factors are activated, boosting cell growth, protein synthesis, and reducing autophagy [187,190].

#### 2.3.12. Caspases

Caspase 1 is elevated in ADPKD kidneys, and once activated, it accumulates interleukin-1β and interleukin-18 in the cystic fluid, presumably through inflammasome activation [191]. In ADPKD, caspases 3, 7, 8 and 9 mediate apoptosis, with “executioner” caspase 3 largely affecting cell morphology and DNA fragmentation and caspase 7 affecting cell viability. Together, caspases 3 and 7 mediate pro-apoptotic loss of mitochondrial membrane potential [192]. Caspases 3 and 7 are proteolytically cleaved by “initiator” caspases 8 and 9 [193].

#### 2.3.13. Bicaudal C

Bicaudal C, abbreviated BicC in Drosophila, Bicc1 in mice and BICC1 in humans, is an RNA-binding protein and translational regulator first identified in Drosophila as a maternal factor directing anterior–posterior patterning in the oocyte and embryo [171,194,195,196,197]. BicC is a key developmental regulator with high conservation extending throughout the entire protein length [197,198]. In both vertebrates and flies, *BICC1/Bicc1/BicC* mutations typically produce renal cysts [128,170,199,200,201,202]. Linking BicC to at least ADPKD-type cystic pathology, *PKD1* and *Pkd1* mutations cause *BICC1*/*Bicc1* loss-of-function, respectively, in humans and mice [128,203]. Thus, *PKD1/Pkd1* loss-of-function phenocopies *BICC1/Bicc1* loss-of-function. Kidneys from *Bicc1^−/−^* mice exhibited downregulated *Pkd2* mRNA compared to wildtype, likely mediated by a micro-RNA (*miR*) *17* binding site in the 3’ untranslated region (UTR); hence, Bicc1 may oppose *miR17*-dependent inhibition of *Pkd2* expression [202]. BICC1 interacts with human polycystins in vitro [204]. In the fly renal tubule, BicC represses the translation of *myc* mRNA [128]. *BicC* mutations cause Myc protein over-expression, mTOR activation, and cystic growth [128]. Despite only beginning to understand its function, BicC/Bicc1/BICC1 is emerging as a new developmental regulator of pathways in the renal tubule relevant for PKD-like renal pathology.

#### 2.3.14. Outlook on Factors Implicated in ADPKD 

Numerous factors are dysregulated in the ADPKD-type cysts, many of which fuel cell growth and division, support the underlying heightened metabolism, and alter cell polarity, communication, and adhesion. In addition, increased oxidative stress, compromised cell death and tissue remodeling contribute to the slow, progressive erosion of renal function, frequently leading to long-onset ESRD. Because these many factors appear interdependent and connected in cellular networks, the effects of specific mutations likely combine with the functional properties of the natural individual variants of their interacting partners to contribute to the observed variable phenotypic presentations. In addition to the genetically defined components, environmental factors such as diet, toxins, and the intestinal microbiota may uniquely modify the PKD phenotypic manifestation [205].

**Table 2:** Factors contributing to ADPKD and potential Drosophila orthologs.

Molecular Trait	Increased/Activated/GOF or Decreased/Inhibited/LOF	Functions	Drosophila Ortholog
PC1, PC2 *Polycystins*	LOF ^6^	Transmembrane proteins. A subset of the cellular pool interacts to form a complex. Reduces adenylate cyclase activity [101,103]	PC1: none. PC2: *Pkd2*, a.k.a. ^8^ *Almost there* (*Amo*)
MYC	GOF ^7^ [100]	Cell growth, proliferation, and death, metabolic dysregulation, inflammation, fibrosis [115,116]. Myc O/E ^5^ in the ADPKD renal cystic epithelium [206]	*d-Myc*
mTOR *Mechanistic target of rapamycin*	GOF ^7^ [121]	Nutrient sensing, cell growth and proliferation, autophagy [207]	*mTor*
c-src *Src proto-oncogene*	GOF ^7^ [2,208]	Activated by cAMP-induced PKA, phosphorylates SHC, activates RAS, RAF kinases [150,208]	*Src oncogene at 42A, (Src42A)*; *Src oncogene at 64B* (*Src64B)*
cAMP *Cyclic adenosine monophosphate*	GOF ^7^ [137]	Second messenger, signal transduction. PC1 LOF ^6^ derepressed adenylate cyclase, increased V2R signaling, inhibited Ca^++^-dependent phosphodiesterase, all increasing cAMP [137]	cAMP (molecule)
TNF-α *Tumor necrosis factor alpha*	GOF ^7^ [157]	Cell growth, apoptosis, inflammation. Found in ADPKD cyst fluids [157]. TNFR1 receptor upregulation (*Pkd1* LOF ^6^ renal epithelial cells) [156]. Stabilized TNF-α-TNFR1 interaction fuels a positive feedback loop and TNF-α O/E ^5^ [157]	*eiger* (*egr*)
Canonical Wnt pathway	GOF ^7^ [209]	Affects β-catenin levels, Ca^2+^ dependent cell adhesion and TCF ^3^/LEF ^4^-dependent transcriptional activation [167]. Dysregulated in *Pkd1^−/−^* cells [209]	wingless (wg) pathway
PCP, non-canonical Wnt pathway *Planar cell polarity*	LOF ^6^ [174]	Linked to Hippo signaling in PKD-like cystogenesis. Dysregulated in *Pkd1^−/−^* cells [209]	PCP pathway *(frizzled, fz; disheveled, dsh)*
Hippo pathway	LOF ^6^ [172]	Tumor suppressor regulating organ size, tissue regeneration, and cystogenesis. In carriers of *PKD1* mutations, Hippo loss of suppression disrupts renal tubule development and causes cystic kidneys [173]. Downstream effectors YAP and TAZ contribute to renal cystic pathways [173,176]	Hippo pathway
Notch	GOF ^7^ [111]	Regulates cell proliferation and differentiation	*Notch* (*N*)
Hedgehog	LOF ^6^ [109]	Signaling pathway activated by primary cilia and regulates cell fate [210]	*Hedgehog (hh)*
YAP	GOF ^7^ [173]	Downstream effector of Hippo	*Yorkie (Yki)*
TAZ	GOF ^7^ [211]	Downstream effector of Hippo	*Yorkie (Yki)*
Bioenergetic remodeling	n/a ^9^	Extracellular acidification, aberrant mitochondria [212,213]	n/a ^9^
Integrin-α1	GOF ^7^ [185]	α subunit of heterodimeric αβ membrane receptors at focal and cell–cell adhesions that link the extracellular matrix to the cytoskeleton	*multiple edematous wings (mew, integrin αPS1)*
Integrin-β1	GOF ^7^ [183,184]	β subunit of heterodimeric αβ membrane receptors at focal and cell–cell adhesions that link the extracellular matrix to the cytoskeleton	*Myospheroid (mys, integrin βPS)*
PI3K/Akt *Phosphoinositide 3 kinase, Serine–threonine protein kinase AKT/Protein kinase B*	GOF ^7^	Cell survival, proliferation, growth, metabolism. Dysregulated in *Pkd1^−/−^* tissue: excess upregulated pathways and downregulated PI3K-AKT inhibitors [209]	*d-Akt*
ERK *Extracellular signal-regulated kinase*	GOF ^7^ [2,214]	Activated by MAPKK, central regulator of cell growth, proliferation, apoptosis, adhesion [150]	*d-Erk*
MEK, or MAPKK *Mitogen-activated protein kinase kinase*	GOF ^7^ [140]	Cell growth, proliferation, apoptosis, RAF/MEK/ERK pathway [150]. Dysregulated in *Pkd1^−/−^* cells [209]	*d-Mek* a.k.a. ^8^ *Downstream of Raf* (*Dsor1*)
B-Raf *Serine–threonine protein kinase B-Raf*	GOF ^7^ [145]	Phosphorylates MAPKK/MEK, cell growth, proliferation, apoptosis [150,215]	*Raf*
Ras *Rat sarcoma virus*	GOF ^7^ [216]	Cell growth [150]	*Ras oncogene at 85D* (*Ras85D*)
Vasopressin *AVP*	GOF ^7^ [217]	Anti-diuretic hormone. Elevated AVP ^2^ promotes cystogenesis and induces typical cardiovascular complications [26,218,219]	n/a ^9^ Anti-diuretic function mediated by nonapeptide inotocin (Ino).
V2R *Vasopressin 2 receptor*	GOF ^7^	AVP ^2^ signal transduction and cystogenesis [219]	n/a ^9^
AMPK *Adenosine monophosphate-activated protein kinase*	LOF ^6^ [2,220]	Cell and cystic growth [2,220]	*AMPKa*
EGFR *Epidermal growth factor receptor*	GOF ^7^ [112]	Expressed in the cells of the glomerulus, loop of Henle, distal convoluted tubule and collecting duct (human adult kidney). In ADPKD, EGFR is mislocalized to the apical membrane [150,221]	*Egfr*
JAK/STAT *Janus kinases/Signal transducers and activators of transcription*	GOF ^7^ [2,222]	Cell and cyst growth [2,222]	*hopscotch* (*hop*)
PGE2 *Prostaglandin E2*	GOF ^7^	Osmoregulation [223]. It increases cell proliferation and Cl^−^ secretion in PC1 LOF ^6^ cells [224]	*Cytosolic prostaglandin E2 synthase* (*cPges*)
NF-kB *Nuclear factor kappa B*	GOF ^7^ [157]	Activated by the TNF-α/TNFR1 complex. NF-kB and FLIP regulate both cell survival and death [157]	NF-kB: *Dorsal-related immunity factor (Dif)*; *Relish* (*Rel)*; *dorsal* (*dl*)
NFAT *Nuclear factor of activated T cells*	GOF ^7^ [2,225]	Ca^2+^-regulated transcription factor, renal tubule epithelial cells survival, inflammation, fibrosis. Fuels cystic growth [2,225].	*NFAT*
Caspase 1	GOF ^7^	Inflammasome, apoptosis, pyroptosis. Promotes cyst formation [191]	*Death caspase 1* (*Dcp-1*)
Caspase 3	GOF ^7^	Apoptosis, executioner caspase [193]	*Death-related ICE-like caspase* (*DrICE*)
Caspase 7	GOF ^7^	Apoptosis, executioner caspase [193,226,227]	*Death-related ICE-like caspase* (*DrICE*)
Caspase 8	GOF ^7^	Death-mediated apoptosis [193]	*Death-related ced-3/Nedd2-like caspase* (*Dredd*)
Caspase 9	GOF ^7^	Apoptosis [193,228]	*Death regulator Nedd2-like caspase* (*Dronc*)
Ca^2+^	LOF ^6^	Intracellular signaling [2,53]. Dysregulated in *Pkd1^−/−^* cells [209]	n/a ^9^
TSC1/TSC2 *Tuberous sclerosis complex*	LOF ^6^	mTOR regulator. The PC1 C-terminal tail (CTT ^1^) prevents TSC2 phosphorylation by AKT, which inhibits mTOR [229]	*TSC subunit 1* (*Tsc1/Tuberin*)
PKA *Protein kinase A*	GOF ^7^	Activated by cAMP [230], triggers growth factor expression and promotes cystic fluid secretion [135,150]	*Protein kinase cAMP-dependent catalytic subunit 1, 2, 3* (*Pka-C1, Pka-C2, Pka-C3*)
sFRP4 *Secreted frizzled-related protein 4*	GOF ^7^ [231]	Inhibitor of canonical Wnt signaling, binds to Wnt and Frizzled; sFRP4 expression in IMCD cells plus a V2R antagonist reduced cyst fluid [231]. Progressively elevated SFRP4 expression in *pcy* mice [231]	*Frizzled* (*fz*), *Frizzled 2–4* (*fz2*, *fz3*, *fz4*),
β-catenin	GOF ^7^ [162]	Cell adhesion, signal transduction. Dysregulation/relief of PC1 CTT ^1^-mediated inhibition on β-catenin, promotes cyst formation and development [167,232]. The β-Catenin/TCF ^3^ complex targets the *PKD1* gene [233]	*armadillo* (*arm*)
PDE (PDE1, PDE6) Phosphodiesterase	LOF ^6^	cAMP hydrolysis [234,235]	*Pde1c, Pde6*
Adenylyl cyclase	GOF ^7^	cAMP synthesis [53]	*Adenylate cyclase 1* (*Adcy1*), a.k.a. ^8^ *rutabaga* (*rut*)
RHEB *Ras homolog enriched in brain*	GOF ^7^	Regulator of mTORC1 [229,236]	*Rheb*
GSK3B *Glycogen synthase kinase 3 beta*	LOF ^6^	Regulates cyst expansion, cell proliferation, AVP ^2^-mediated urine concentration by renal collecting duct cells [237]. Regulates adenylate cyclase activity and cAMP synthesis [238]	*shaggy* (*sgg*)
OCD *Orientation of cell division*	LOF ^6^ [172]	Orientation of cell division. Dilating cysts feature OCD LOF ^6^. Dispensable for cyst formation [239]	Spindle positioning and asymmetric cell division pathways, e.g., *inscuteable* (*insc*), *partner of inscuteable* (*pins*), *bazooka* (*baz*, *Par-3*), *Par-6*
Id2 *Inhibitor of DNA binding 2*	LOF ^6^ [155]	Interacts with PC1 and PC2 complex, cell cycle regulation [155]	*Extramacrochaetae (emc)*
BICC1 *Bicaudal C*	LOF ^7^ [128]	Translational regulation, Wnt signaling. Downregulated in *PKD1* and *Pkd1* LOF ^6^ [128,203]. Molecular interaction with PC1, PC2 [204]	*Bicaudal C (BicC)*
AP-1 *Activator Protein 1*	GOF ^7^ [211]	Regulates cell proliferation when PC1 is downregulated [211]	

^1^ CTT (PC1 C-terminal tail), ^2^ AVP (arginine vasopressin), ^3^ TCF (T-cell factor), ^4^ LEF (lymphoid enhancing factor), ^5^ O/E (over-expression), ^6^ LOF (loss-of-function), ^7^ GOF (gain-of-function), ^8^ a.k.a. (also known as), ^9^ n/a (not available).

### 2.4. Ciliary and Metabolic Signature of ADPKD

Several proteins implicated in polycystic kidney diseases are localized at/near the primary cilia. Thus, cilia were initially thought to majorly contribute to renal cysts, and ADPKD, ARPKD and NPH were considered ciliopathies [240,241,242]. However, mutation of either polycystin or ciliary components showed that while impairing cilia caused much milder cystic phenotypes than the *Pkd1* or *Pkd2* loss-of-function alone, double mutation clearly ameliorated the severe *Pkd1* or *Pkd2* phenotype [120]. Thus, cilia appeared to contribute to cyst pathology, being neither necessary nor sufficient for it [243]. The cilium may promote cyst formation through the CDCA signal, which is normally compensated for by polycystins [120,244,245,246]. When polycystin activity drops below a threshold, the CDCA “break” becomes insufficient and polycystic defects manifest [247,248], possibly in context-dependent ways [249]. Although the CDCA remains elusive, a few components identified include the NPH-linked Glis2 protein and the metabolic enzyme asparagine synthetase [246,250,251,252,253,254,255]. Co-localizing with PC2 at the primary cilium of renal epithelial cells, the exocyst is also needed for both cell growth and the cilium [256,257]. The exocyst protein complex (Sec3p, Sec5p, Sec6p, Sec8p, Sec10p, Sec15p, Exo70p, Exo84p) directs exocytic vesicle formation from the trans-Golgi network to the plasma membrane [258]. Mutations in exocyst components may also lead to cystic kidney disease [256,259,260]. Metabolic remodeling is a feature of cystic pathologies and neoplasia, but, at least in the kidney, it does not appear to be the main cystic driver [261,262]. Early transcriptomics studies on resected human ADPKD tissue and cultured cells from ADPKD individuals suggested both individual differences and consensus dysregulation of several pathways, including those supporting cell proliferation and metabolism, signal transduction, renal transport, immune function and inflammation [65,263]. An emerging metabolic signature of PKD portrays altered glucose metabolism consistent with a cancer-like “Warburg effect” (or aerobic glycolysis) and glutamine utilization [98,250,254,264,265,266,267]. Coherently, administration of glucose analogs and ketosis appeared to lessen pathology [268,269]. Amino acids asparagine, arginine, tryptophan, methionine and lipid metabolism are also affected in ADPKD cells [250,254,263,264,270,271,272,273,274,275,276]. Other critical components appear to be the micro-RNA *miR-17* upregulation and peroxisome proliferator-activated receptors (PPAR) transcription factors [274,275,277,278]. Several attempts at therapeutic targeting of such factors are being tested at the pre-clinical and early clinical stage [279] (below, Table A1). ADPKD also reduced oxidative phosphorylation in several PKD models and patient cells [250,263,270,280,281,282]. Consistent with these defects, dysmorphic and fragmented mitochondria, impaired mitochondrial respiration, and enhanced oxidative stress were observed in murine *Pkd1* mutant cells and PKD [280,283,284,285,286]. A fraction of human PC1 and PC2 was found at the contact between the endoplasmic reticulum and mitochondria [283]. Fittingly, PC2 regulates mitochondrial transporter SLC25A25 [287]. Proteolytic C-terminal PC1 cleavage exposes a cryptic mitochondrial localization sequence [284]. Finally, the polycystin C-terminal fragment regulates the nicotinamide nucleotide transhydrogenase, which is likely functionally relevant because fragment over-expression compensated for *Pkd1* defects [288].

### 2.5. ADPKD Treatment

Likely reflecting its unresolved phenotypic complexity, ADPKD is incurable, and most treatments manage the degrading kidney function, cardiovascular complications, inflammation, pain, and modifiable risk factors (Table 3). The PKD Foundation [289] suggests that PKD-affected individuals manage blood pressure, restrict dietary sodium, and, unless there are hyponatremic risks, increase fluid intake to at least 3 L daily. Higher sodium excretion elevates risk of kidney growth and eGFR decline; high fluid intake reduces risk of nephrolithiasis (kidney stones) and quells vasopressin levels, opposing pro-cystic vasopressin signaling. Hypertension in PKD may be caused by direct and compensatory physiological effects, including abnormal ciliary and vascular function, cardiovascular dysfunction, activation of the renin–angiotensin–aldosterone system, or potentially intrarenal ischemia in the cystic kidneys [290]. The renin–angiotensin–aldosterone system contributes to regulating blood volume and pressure and balancing fluid and electrolytes. Blood pressure is managed through typical lifestyle modifications (limiting salt and caffeine intake, exercising regularly and avoiding or ceasing smoking) and pharmacologically using inhibitors of the renin–angiotensin–aldosterone system [290,291]. Tolvaptan, a repurposed selective V2R antagonist, was approved worldwide between 2014 and 2018 for PKD use to balance fluids and reduce cyst growth [144,217,292,293]. Tolvaptan only benefits a few patients with specific presentations, may be hepatotoxic, and its long-term efficacy is unknown [294]. Early results with first-generation mTOR inhibitors rapamycin [123] and rapamycin analogs (rapalogs), e.g., everolimus, showed encouraging reduction in the cystic index and total kidney volume, two indices of PKD severity [125,295]. However, the long-term efficacy of mTOR inhibition has been unsatisfactory, even in combined systems that target the renal tubules and cysts preferentially to improve drug tolerability [296,297]. mTOR inhibitors were found to disrupt renal epithelial cell proliferation, albeit transiently, which limits therapeutic applications [123,124,298]. Octreotide, an inhibitor of cAMP production, has also been used [299]. Long-acting release octreotide slowed kidney growth and decline towards ESRD in later-stage ADPKD and was well tolerated [300,301]. However, a recent meta-analysis found similar reduction in cystic kidney growth and good tolerance, but no apparent effects on eGFR [302]. Long-acting release octreotide was approved in Italy in 2019 for use in advanced ADPKD patients with stage 4 CKD and high risk for ESRD [303]. Several clinical trials have been performed and are ongoing for ADPKD (Table A1). Due to the dearth of PKD therapeutics, several strategies based on renormalizing the activity of pathways dysregulated in PKD have been and are being studied and were reviewed recently [304,305]. However, the apparent multi-factorial nature of ADPKD is expected to reduce the efficacy of therapeutic interventions targeting specific aspects and disease pathways and may need to be addressed with emerging combination therapies [64,306] that may be designed to address individualized network disruptions and to control renal degeneration in pediatric patients who inherited pathological mutations. Moreover, with chronic ADPKD, long-term compensatory mechanisms may reduce the effectiveness of corrective therapies over time. Once renal function has become insufficient, hemo- and peritoneal dialysis are used before resorting to transplantation. In both cases, ADPKD patients have better outcomes and fewer complications than non-PKD patients with ESRD [64,307,308]. For hemodialysis in specialized centers, surgically placed vascular access enables dialysis machines to perform blood filtration in about four hours every other day. Peritoneal dialysis, in contrast, uses a catheter to fill the peritoneal cavity with a dialysate fluid that draws catabolites and fluids from the blood and through the peritoneal membrane and is then drained. Slower peritoneal dialysis must be carefully managed to prevent infections but can be performed at home at nighttime, for less life disturbance. Although lifesaving, both dialysis protocols severely disrupt the quality of life of the ADPKD-affected individuals and their families. Thus, more precise mechanistic knowledge of the PKD pathology is urgently needed to design effective and non-toxic therapeutics suited to the chronic nature of PKD.

## 3. ARPKD

### 3.1. ARPKD Phenotypes, Genetics, and Pleiotropy

ARPKD (OMIM 263200), a rare and prototypical hepato-renal fibrocystic disease, displays a carrier frequency of 1 in 70 and affects an estimated 1:20,000 live births [2,314,315]. ARPKD is comparatively more severe than ADPKD and is typically diagnosed prenatally because of enlarged hyperechogenic kidneys and congenital hepatic fibrosis [316]. ARPKD features an ill-defined kidney cortex and medulla, multiple tiny cysts dotting the renal distal tubules and collecting ducts with typical fusiform dilations, and usually presents in utero, perinatally, or in infancy [2,317,318,319,320]. The hyperechogenic enlarged kidneys typical of ARPKD are detected antenatally and may be associated with oligo- or anhydramnios (scarce or absent amniotic fluid, respectively) [315,321] that can produce the head malformations of Potter sequence Type I [322]. ARPKD also causes congenital hepatic fibrosis, dilated bile ducts and portal hypertension [320] and becomes lethal for 30% of newborns [2]. Neonates with ARPKD die in their first six weeks because of respiratory insufficiency and infections, and 70% of the survivors develop ESRD by age 20 [317,318,319]. Like ADPKD, ARPKD presents with hypertension and extrarenal manifestations with partially overlapping organ involvement (Figure 1) [43,317,318,319]. Except for the full penetrance of the cystic phenotype, data on the frequency of renal and extrarenal phenotypes often show discrepancies and small target populations (Table 4A,B). Mild forms of ARPKD may resemble ADPKD and vice versa; strong forms of ADPKD may phenocopy ARPKD [323]. Mutations in the *PKHD1* gene largely account for the ARPKD cases [2]. The *PKHD1* gene encodes fibrocystin, also called polyductin, another transmembrane protein [2,6]. The disease-associated *PKHD1* genetic variants are distributed throughout the gene, seemingly without mutational hot spots, although a missense mutation in exon 3 accounts for 10–15% of all *PKHD1* mutations [67]. While the genotype–phenotype relationships remain relatively undefined, mutations producing truncated proteins are frequently found in severe perinatal forms, and specific missense mutations seemingly associate with higher severity [9,318,324,325,326,327]. Although *PKHD1* mutations account for most of the ARPKD cases, five homozygous mutations in the gene *DAZ-interacting protein 1-like* (*DZIP1L*) on the 3q22.1-q23 chromosome were found in five ARPKD patients [324]. Furthermore, very-early-onset PKD [328,329,330,331], HNF1B nephropathy [332], and both DZIP1L [333,334] and CYS1 [332]-driven kidney disease can phenocopy ARPKD. Genetic and/or environmental modifiers are also expected because variable phenotypes can be observed in family members who inherited the same mutation [3,325]. A single copy of *PKHD1* mutated variants may also act as a modifier of the *PKD1*-induced phenotype [330]. Fibrocystin has developmental functions, likely directing tubulo- and organo-genesis in the embryo and the terminal differentiation of epithelia in the adult. In the embryo, it is found in the mesonephric tubules, ureteric bud, adrenal cortex, hepatocytes, primordial gut, heart, neural tube and bronchi [3,325]. In adults, fibrocystin is normally expressed in renal collecting ducts and hepatic and pancreatic ducts, and its inactivation causes PLD [334]. These observations yielded the hypothesis that defects in the terminal differentiation of epithelia may underlie and/or contribute to ARPKD.

### 3.2. Factors Implicated in ARPKD and Development

Like ADPKD, several factors have been found to contribute to ARPKD, including evolutionarily conserved regulators of cell growth and apoptosis. Below, in Table 5, and Figure 2 is a summary of their developmental and renal functions, evolutionary conservation, and involvement in cystic kidney diseases.

#### 3.2.1. Fibrocystin

The *PKHD1* mRNA contains 87 alternatively spliced exons. The longest transcript encodes a 4074 amino acid protein product. Fibrocystin has an extensive extracellular N-terminal domain, a single transmembrane region, and a short C-terminal cytoplasmic tail, resembling the Notch developmental receptor [6,323,347,348,349]. Fibrocystin is found in the renal tubule at the apical side of the cells of the thick ascending Henle’s loop, the proximal convoluted tubule, the collecting duct [348,349,350], and the pancreatic and bile ducts [6,347]. In the renal tubular epithelium, fibrocystin is found in the primary apical cilia, basal body, apical membranes and nucleus [347,351,352]. Moreover, the urinary extracellular vesicles also contain fibrocystin [350,353]. In cultured human cells, fibrocystin localizes to the basal body and primary cilium and, in dividing cells, to the centrosome and mitotic spindles [6,324,325,347,349,354]. Consistently, *Pkhd1* knockout mice have dysmorphic cilia and the *PCK* rat, an ARPKD model, displays ciliary defects in the cholangiocytes of the hepatic bile ducts [355,356,357]. Fibrocystin may regulate intracellular Ca^2+^ stores [349,358]. Like Notch, following binding to an unknown ligand, fibrocystin is first proteolytically cleaved to yield two fragments linked by a disulfide bond, and, upon intracellular Ca^2+^ increase, cleaved again to release the cytoplasmic tail that translocates to the nucleus and regulates downstream target genes [359,360]. However, it is unclear if the similarity between fibrocystin and Notch extends to their function, besides having similar proteolytic maturation. The soluble extracellular portion is shed in the urine or, possibly, in extracellular vesicles together with PC1 and PC2. Extracellular vesicles may function in intraluminal or “urocrine” signaling that is thought to coordinate epithelial function and have a developmental role. However, neither of these roles has been precisely defined [323,348,350,353,361,362,363]. A typical urine 77-peptide signature was found in ARPKD cases [320] but not in other renal conditions that phenocopy ARPKD e.g., very-early-onset PKD [329,330,331,332], HNF1B nephropathy [333], and DZIP1L- [324,364] and CYS1- [333] kidney disease. Besides its diagnostic value, this signature may also hint at ARPKD mechanisms; for example, peptide fragments from Ca^2+^ binding proteins weigh in on a Ca-related function for fibrocystin [320]. Studies of the tractable hepatic bile ducts have shown that in both human ARPKD and murine *Pkhd1^−/−^* cells in vivo, endogenous fibrocystin-containing extracellular vesicles were unable to fuse with the ciliary membrane, suggesting that fibrocystin may be involved in vesicular fusion [353,365]. Seemingly, *Pkhd1* mutations must be associated with polycystin or other cyst-inducing mutations to yield a cystic phenotype in mice [366,367]. Similarly, the human and mouse fibrocystin have divergent intracellular portions, and *Pkhd1^−/−^* mice do not fully phenocopy human ARPKD [368,369,370]. This is interpreted as a manifestation of unique renoprotective pathways in mice. Part of the fibrocystin intracellular domain is localized to the mitochondria through a mitochondrial localization sequence that may be functionally relevant because renal tubule cells from *Pkhd1^−/−^* mice had defective mitochondria [213,371]. Fibrocystin may interact molecularly and genetically with PC2 [349,356,372], although their physical interaction has been called into question [350]. Direct interaction with PC1 has not been shown [367], although a cooperative function in cilia has been proposed [268]. The fibrocystin C-terminal fragment is thought to act in several ways. First, together with the PC1 C-terminal fragment, it dampens the CDCA signal preventing mitochondrial involvement; second, it protects mitochondria; third, in the nucleus it binds to several promoters (e.g., human *MYC* and *SRC* and mouse *Myc* [373]); fourth, it regulates ubiquitination through the adaptor and regulator of HECT-type ubiquitin ligases, the Nedd4 Family Interacting Protein 2 (NDFIP2) protein. Reduced ubiquitination, in turn, promoted pro-cystogenic transforming growth factor beta (TGF-β) signaling, fibrosis, disruption of sodium channels, RhoA expression and cytoskeletal organization [268,373,374,375]. Interestingly, despite the fibrocystin C-terminal domain binding to both human and murine promoters, MYC over-expression was only observed in human cells [375], possibly because of species-specific differences in the temporal expression of the *MYC/Myc* regulator CYS1/Cys1 [375]. Similarly, the SRC interaction dysregulated STAT3 [373] and PI3K/Akt/mTOR signaling [376] only in human cells. Ubiquitination and proteostatic pathways were disrupted in *Pkhd1* loss-of-function murine cells, without causing the cystic phenotype [377,378]. Despite mechanistic differences, the mitochondrial connection may be relevant for the metabolic remodeling observed in ARPKD. Fibrocystin knockdown in cultured renal cells suggested its implication in cell adhesion and development [379,380]. In mice (inner medullary collecting duct) IMCD3 cells, fibrocystin loss-of-function prevented E-cadherin and Zonula Occludens-1 (ZO-1) localization at the cellular junctions, impaired integrin-mediated adhesion, and affected cell polarity, survival and ERK signaling, all pathways critical for tubular morphogenesis [381,382]. In Madin–Darby canine kidney (MDCK) cells, fibrocystin loss-of-function also altered adhesion and invasiveness, although the precise outcomes may depend on experimental conditions [380,383]. Although the mechanism remains unknown, these findings support a possible developmental role for fibrocystin and involvement in some of the same pathways as the polycystins. Both AD and ARPKD display fibrosis (Figure 1). Cultured cholangiocytes from ARPKD suggested that fibrosis may be induced by abnormal secretion of interleukin-8 and connective tissue growth factor and dysregulated immune function [384,385].

#### 3.2.2. DZIP1L

Rare mutations in the gene encoding the DAZ-interacting zinc finger protein 1 like (DZIP1L) and homozygous mutations have been found in cases presenting as ARPKD when a *PKHD1* mutation is absent [324,364]. DZIP1L is a zinc-finger protein that localizes to centrioles and the distal end of basal bodies and is involved in the localization and function of ciliary proteins like PC1, PC2, and fibrocystin. DZIP1L interacts with Septin 2, a factor needed for the integrity of the periciliary diffusion barrier at the ciliary transition zone [324,364]. While the human subjects with DZIPL1 mutations typically presented with kidney defects, *Dzipl1* mutant mice and zebrafish *dzipl1* morphants exhibited widespread defects [324]. Consistent with ciliary regulation of Hedgehog signaling, *Dzip1l^wpy/wpy^* mutant mice developed four-limb polydactyly, craniofacial defects such as cleft palate and bilateral cleft lip, and in 67% of the animals, also internalized eyes [324]. The *Dzip1l^wpy/wpy^* mice in a C57BL/6:C3H mixed background showed progressive cystic kidney phenotypes with near-cortical cysts forming during late embryogenesis [324]. Once crossed to an outbred CD1 background, the animals displayed near-cortical cysts during late embryogenesis, and the phenotype became more severe as the mice aged, including extrarenal manifestations atypical for human ARPKD, e.g., extra bile ducts, cleft palate, bilateral cleft lip, and abnormal and internalized eyes [324]. Similarly, morpholino-mediated *dzipl1* knockdown in zebrafish produced highly penetrant otolith defects and 0–10% renal defects [324]. These results suggest that human *DZIPL1* mutations may be less consequential for ciliary functions than mutating its murine orthologs, possibly because of ciliary functional redundancy from DZIP1 [386]. Consistent with species-specific functional differences, cyst distribution in the *Dzipl1^wpy/wpy^* mice differed from human ARPKD, with the former affecting collecting ducts and proximal tubules and the latter affecting collecting ducts and predominantly distal tubules [324,387]. In *Pkhd1* mice, different alleles produce effects in different tubular regions [324,353,355,388], suggesting that the tubular regions may be differentially sensitive to the effects of specific mutations.

#### 3.2.3. Signaling and Developmental Pathways

Several developmental signal transduction pathways central to regulating cell size, division, and survival are similarly activated in AR and ADPKD, e.g., cAMP, MYC, mTOR, and caspases (Table 5) [2,228,389]. In ARPKD, phospho-mTOR, phospho-S6K, 4EBP1, and AKT were all over-expressed in the cystic epithelium [389,390]. In ARPKD patients with liver involvement, phospho-S6K was strongly expressed in the parenchymal hepatic cells and bile ducts, while the control healthy tissue had no phospho-S6K staining, which indicates mTOR overactivation in the cyst-lining renal cells and the bile duct epithelium [390]. Like ADPKD and thought to be linked to the PKD ciliary component, ARPKD disrupts WNT signaling, both canonical (through WNT4, WNT9B) and non-canonical (through WNT5A, WNT9B) [161,391,392,393,394]. All these factors also function in development: the mouse WNT4 orthologs Wnt4 and Wnt9b are key regulators of tubulogenesis and mesenchymal-to-epithelial transition in the murine kidney [395,396,397,398]; Wnt5a regulates podocyte development, affecting the cytoskeleton, slit diaphragm, and cell motility [399]; Wnt9b also regulates PCP, cell orientation and proliferation in renal tubule morphogenesis [397,398]. Based on sequence conservation, a recent hypothesis posits that *PKHD1*, only found in the genome of terrestrial vertebrates, is evolutionarily recent, and *DZIP1L* is more ancestral and might reflect physiological adaptations underlying the transition from aquatic to terrestrial environments [316]. In this view, the functional differences and distinct requirements to achieve observable phenotypes in human and murine renal tissues may reside in the fibrocystin recent ancestry [316].

**Table 5:** Factors contributing to ARPKD and potential Drosophila orthologs.

Molecular Trait	Increased/Activated/GOF or Decreased/Inhibited/LOF	Function	*Drosophila* Ortholog
*PKHD1* *Fibrocystin/Polyductin*	LOF ^3^	May regulate intracellular Ca^2+^ stores [349,358]	n/a ^1^
MYC	GOF ^2^ [100]	Cell growth, proliferation, and death, metabolic dysregulation, inflammation, fibrosis [115,116]	*d-Myc*
mTOR	GOF ^2^ [389]	Nutrient sensing, cell growth and proliferation, autophagy [207]	*mTor*
Src	GOF ^2^ [373]	Activated by cAMP-induced PKA, phosphorylates SHC, activates RAS, RAF kinases [150]	*Src 42A*, *Src 64B*
cAMP	GOF ^2^ [140]	Second messenger and signal transduction	cAMP (molecule)
TNF-α	GOF ^2^ [153,400]	Cell growth, apoptosis, inflammation	*Eiger* (*egr*)
Wnt	GOF ^2^ [393,401]	Affects β-catenin levels, Ca^2+^ dependent cell adhesion and TCF ^4^/LEF ^5^-dependent transcriptional activation	*Wingless* (*wg*)
Hippo	LOF ^3^ [401]	Downstream effectors YAP and TAZ contribute to renal cystic pathways [173,176]	*Hippo* (*hpo*)
Notch	GOF ^2^ [111]	Regulates cell proliferation and differentiation	*Notch* (*N*)
Hedgehog	Hypothesized GOF ^2^ [402]	Signaling pathway activated by primary cilia and regulates cell fate [210]	*Hedgehog* (*hh*)
YAP	GOF ^2^ [401]	Downstream effector of Hippo	*Yorkie* (*yki*)
TAZ	GOF ^2^ [401]	Downstream effector of Hippo	*Yorkie* (*yki*)
Bioenergetic remodeling	n/a ^1^	Higher cellular O_2_ consumption, extracellular acidification, mitochondrial dysmorphia [212,213]	n/a ^1^
STAT3	GOF ^2^ [373]	Pro-proliferative transcription factor	*Stat92E*
DZIP1L	LOF ^3^ [324]	Implicated in protein entry into the cilia [403]	*Dzip1* (DAZ interacting zinc finger protein like 1)
Septin 2	--	DZIP1L interactor [326]	*D-Sept2*
CYS1	LOF ^3^ [404]	*MYC* regulator [375]	*Cys*
Apoptosis	GOF ^2^ [228]	Increased apoptosis in collecting ducts (some models) [228]	Apoptosis pathways (e.g., *Reaper*, *Hid*, *Grim*), caspases (e.g., *Dcp-1*, *DrICE*, *Dredd*)

^1^ n/a (not available), ^2^ GOF (gain-of-function), ^3^ LOF (loss-of-function), ^4^ TCF (T-cell factor), ^5^ LEF (lymphoid enhancing factor).

### 3.3. Metabolic Signature of ARPKD

Like ADPKD, ARPKD also displays metabolic reprogramming including glutamine dependence, *miR-17* upregulation, abnormal lipid metabolism and reduced oxidative phosphorylation [98,274,277,278,371,405]. Like PC1, fibrocystin is also cleaved exposing a mitochondrial localization sequence in its C-terminal fragment [213]. *PKHD1* mutations in (human) HEK-293 cells featured misshapen mitochondria and impaired respiration that could be rescued by over-expressing the C-terminal fragment [213,371].

### 3.4. Outlook on Factors Implicated in ARPKD

Similar to ADPKD, ARPKD presents renal cysts and may phenotypically resemble the most severe forms of ADPKD [316]. Fibrocystin and the polycystins are found at several of the same subcellular locations at least some of the time, e.g., the cell junctions, mitochondria, cilia, and may be involved in extracellular vesicle signaling. Several downstream factors are shared between ARPKD and ADPKD (Figure 2, Table 2 and Table 5). However, *PKHD1* may be a relatively modern gene with partial functional overlap with *DZIP1L* and potential neofunctionalization, which may explain some of the typical differences and organ involvement in human and animal models of ARPKD and ADPKD.

### 3.5. ARPKD Treatments

Like ADPKD, ARPKD is incurable. Symptom management is typically tailored to the patient’s young age and degree of disease progression (Table 6). Dialysis within the first days of life well into childhood can be required for neonates with oliguria or anuria [314,406,407,408]. Common dehydration and insufficient weight gain are countered by supplemental feeding or fluid therapy [328] until the 1-year-old milestone is reached, when the risks of transplanting a kidney (and liver if needed) typically decrease. Pulmonary complications and potentially severe intolerance are managed conservatively (Table 6) [328]. A few treatment options are under investigation, including use of the V2R antagonist and ADPKD-approved drug tolvaptan in children with a high risk of ESRD [215,217,328,409]. A Phase 1/2 multicenter clinical trial tested multi-kinase inhibitor tesevatinib to slow the progression of hepatobiliary and renal cystic disease [410]. Because of the limited therapeutic options for ARPKD, it is urgent to identify new effective therapeutics.

## 4. NPH

### 4.1. NPH Phenotypes, Genetics and Pleiotropy

**Figure 3:**
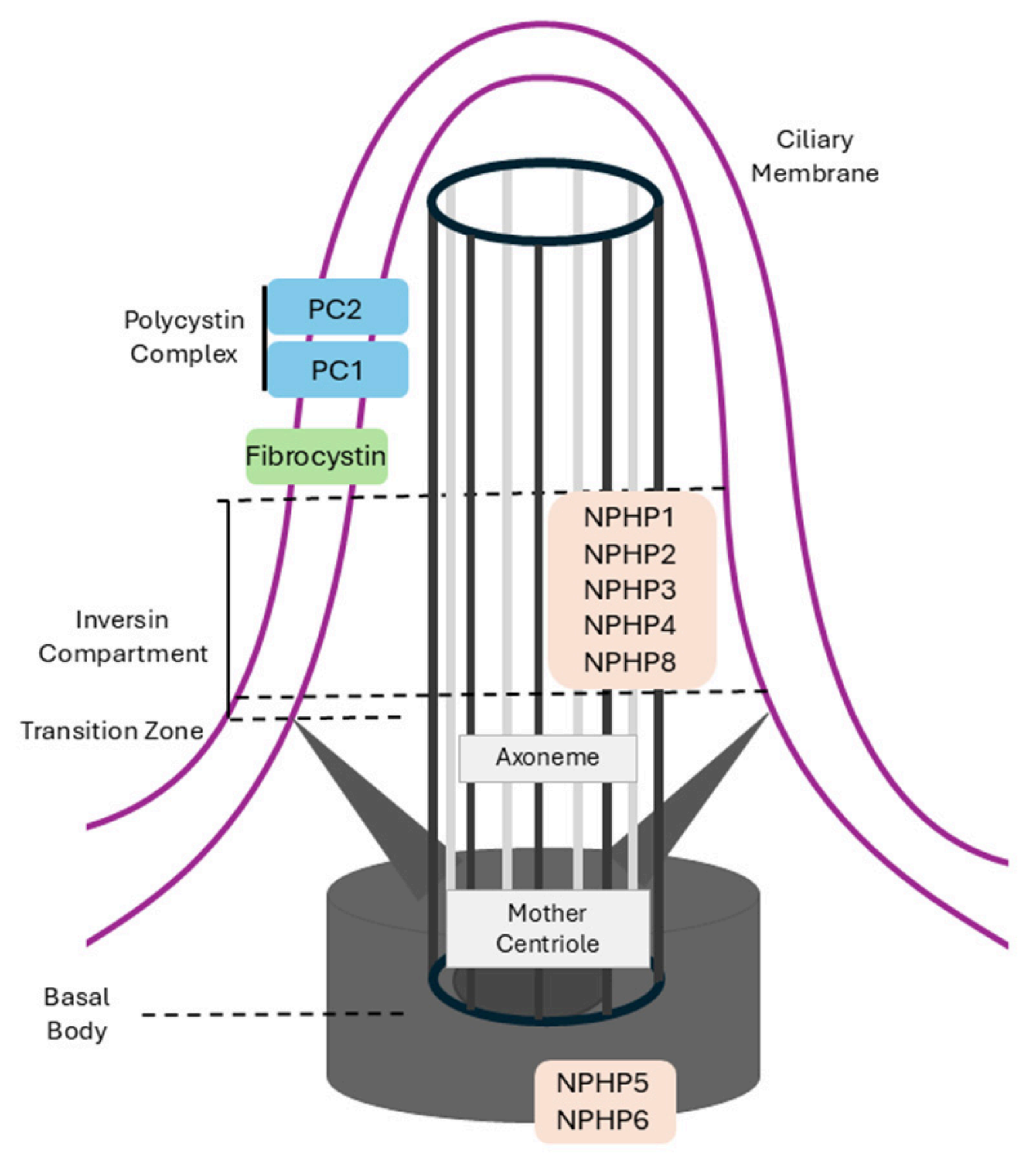
**Ciliary structure and localization of ADPKD, ARPKD and NPH factors within the cilium**.

Autosomal recessive NPH is a frequent cause of ESRD linked to mutations in more than 26 *NPHP* genes encoding proteins collectively called nephrocystins that produce infantile, juvenile and adolescent onset forms [79,412,413,414,415]. The rare, more severe infantile form causes kidney failure by age 3, while the most frequent juvenile form leads to kidney failure at about 13 years of age [79,80]. NPH has variable incidence and occurs at estimated frequencies of 1 in a million in the United States, 1 in 50,000 in Canada, and 1 in 61,800 in Finland [416,417,418,419,420]. The frequency of the rarer infantile form is unknown, per the Orpha.net database. The most common mutation causing about 20% of the isolated NPH cases is a homozygous *NPHP1* deletion [421,422], followed by hits in ten other genes, amounting together to ~3% of the NPH cases [412]. About 70% of NPH-affected individuals harbor mutations in *nephrocystin* genes, and the remaining have unknown cause [241]. Distinct from ADPKD and ARPKD, NPH produces kidney cortico-medullar cysts, tubulointerstitial fibrosis, and compromised urine concentration with polyuria and polydipsia at different frequencies (Table 7A) [79,80]. At the onset, cysts are microscopic and cluster at the distal tubule and collecting ducts [423], but as the disease progresses, cysts grow in both number and size and may occupy the entire kidney medulla [415]. Clinically, 15–20% of NPH-affected individuals experience extrarenal symptoms, including retinal degeneration, bone defects, liver fibrosis, cerebellar vermis aplasia and oculomotor apraxia (Figure 2, Table 7B) [143]. Mutations in the *NPHP* genes cause syndromes in the Joubert–Meckel–nephronophthisis spectrum (Table 8, [424]). Several NPHP proteins are linked to ciliary intraflagellar transport, the centrosome and PCP (Figure 3, Table 8) [415,425,426]. In contrast, NPHP20/MAPKBP1, NPHP1L/XPNPEP3, and NPHP2/SLC41A are not found at the cilia [427,428,429]. Furthermore, multiple NPH-associated genes such as *CEP290*, *NEK8*, *SDCCAG8*, *ZNF423*, *CEP164* and *MAPKBP1* were linked to the DNA damage response (Table 8) [429,430,431,432,433]. Juvenile NPH type 1 is primarily caused by *NPHP1* mutations (Table 8) [434]. *NPHP2/INVS* mutations cause early-onset ESRD by age 5, possible situs inversus and cardiac abnormalities, potential antenatal oligohydramnios and, rarely, retinitis pigmentosa [435]. Mutations in human *NPHP3* produce adolescent and infantile NPH, liver fibrosis, retinitis pigmentosa, and Meckel–Gruber syndrome [413]. *NPHP4* mutations account for ~2% of NPH cases [436,437]. Truncating mutations of *NPHP5/IQCB1* cause early-onset retinitis pigmentosa and Senior–Løken syndrome 1 (or renal–retinal syndrome, OMIM#2669000, NPH plus retinitis pigmentosa) [438]. *NPHP6* mutations cause Joubert syndrome (or cerebello-oculo-renal syndrome, OMIM#213300, [439,440]). Note that about 30% of the genes implicated in Joubert Syndrome also cause NPH [441]. Patients with *NPHP8/RPGRIP1L* missense mutations typically have partial growth deficiency, polydactyly, scoliosis, and pituitary agenesis [442]. *NEK8* mutations cause NPH type 9 [419]. In five cases, renal cystic dysplasia was caused by missense mutations and hypodysplasia (congenitally small kidneys) by nonsense mutations, together with situs inversus, cardiopathy and bile duct paucity [175]. One 3-year-old patient with a *NEK8* missense mutation developed infantile NPH, while two other patients had one additional homozygous *NPHP5/IQCB1* mutation and retinitis pigmentosa [419]. Individuals with mutations in *NPHP11/MKS3/TMEM67* present a wide spectrum of NPH-like conditions with ciliopathies such as liver disease, Joubert Syndrome, Senior–Løken Syndrome, and Meckel–Gruber Syndrome [443]. A study of 62 individuals with NPH found that five harbored *MKS3/TMEM67* mutations and four patients out of 120 displayed both Joubert Syndrome and NPH [443,444]. All these distinct and similar manifestations highlight the NPH phenotypic variability. In contrast, *NPHP1L/XPNPEP3* deficiency yielded a renal histopathology consistent with, yet distinct from, NPH. One patient displayed hypertension, cardiomyopathy, renal failure and seizures [427], and two children with biallelic *NPHP1L/XPNPEP3* splicing mutations presented early-onset renal insufficiency and progressive cardiomyopathy [445].

**Table 7:** (**A**) Renal-associated manifestations and incidence of NPH, ranked by incidence. (**B**) Extrarenal manifestations and incidence of NPH, ranked by incidence.

Symptom	Frequency
**(A, renal)**	
Potentially cystic chronic tubulointerstitial nephropathy	100% [415,416,422,423,435,446,447,448]
Cysts	100% (*n* = 976) [449]; ~70% (incidence = n/a ^2^) [450]
Absent kidney enlargement	No/slightly decreased kidney size at presentation in juvenile and adolescent forms [447]; present in infantile form [435]
Polyuria	>80% (incidences n/a ^2^) [449]; 63% (*n* = 60, *NPHP1* mutations); 36% (*n* = 92, non-*NPHP1* mutations) [451]
Secondary enuresis	>80% (*n* = 6) [452]; 8% (*n* = 24) [453]
Deficient urinary concentration	40% (*n* = 60, *NPHP1* mutations) [451]; 26% (*n* = 92, non *NPHP1* mutations); >80% (incidence n/a ^2^) [449]
Proteinuria	32% (*n* = 60, *NPHP1* mutations); 39% (*n* = 92, non-*NPHP1* mutations) [451]
Atrophic kidneys	35% (*n* = 23) of NPH-related ciliopathies [453]
ESRD ^1^	0.9% of ESRD ^1^ cases carried an *NPHP1* deletion (*n* = 1250) [454]
Interstitial fibrosis	Incidence n/a ^2^ [447]
**(B, extrarenal)**	
Polydipsia	>80% (incidence n/a ^2^) [449]
Anemia	68% (*n* = 152), 88% (*n* = 60, *NPHP1* mutations), 55% (*n* = 92, non-*NPHP1* mutations) [451]
Visual impairment	23% (*n* = 60, *NPHP1* mutations); 41% (*n* = 92, non-*NPHP1* mutations) [451]
Developmental delay	19% (*n* = 60, *NPHP1* mutations); 40% (*n* = 92, non-*NPHP1* mutations) [451]
Hepatosplenomegaly	3% (*n* = 60, *NPHP1* mutations); 29% (*n* = 92, non-*NPHP1* mutations) [451]
Growth retardation	28% (*n* = 60, *NPHP1* mutations), 32% (*n* = 92, non *NPHP1* mutations) [451]
Ataxia	8% (*n* = 60, *NPHP1* mutations); 21% (*n* = 92, non-*NPHP1* mutations) [451]
Astigmatism	17% (*n* = 60, *NPHP1* mutations); 12% (*n* = 92, non-*NPHP1* mutations) [451]
Cholestasis	10% (*n* = 92, non-*NPHP1* mutations) [451]
Cerebellar vermis aplasia	3% (*n* = 60, *NPHP1* mutations); 20% (*n* = 92, non-*NPHP1* mutations) [451]
Thrombopenia	3% (*n* = 60, *NPHP1* mutations); 11% (*n* = 92, non-*NPHP1* mutations) [451]
Esophageal varices	8% (*n* = 92, non-*NPHP1* mutations) [451]
Seizures	7% (*n* = 60, *NPHP1* mutations); 8% (*n* = 92, non-*NPHP1* mutations) [451]
Liver fibrosis	6.2% (*n* = 440 NPHP patients) [420]
Retinal degeneration/retinitis pigmentosa (RP)	5% (*n* = 60, *NPHP1* mutations); 18% (*n* = 92, non-*NPHP1* mutations) [451]
Hepatic pruritus	2% (*n* = 60, *NPHP1* mutations); 2% (*n* = 92, non-*NPHP1* mutations) [451]
Oculomotor apraxia	1% (*n* = 976, *NPHP1* mutations) [449]
*Situs inversus*	Incidence n/a ^2^ [415]
Secondary hyperparathyroidism	Incidence n/a ^2^ [415]

^1^ ESRD (end-stage renal disease) ^2^ n/a (not available).

**Table 8:** Functions of frequently mutated NPH genes and potential Drosophila orthologs.

Gene and Protein	Function(s)	LOF Phenotypes, Clinical Outcomes, [OMIM# Disease Phenotype, *Gene Entry]	Drosophila Ortholog
*NPHP1* Nephrocystin-1	Interacts with proline-rich tyrosine kinase 2 (Pyk2) tensin, focal adhesion docking and extracellular matrix spreading protein p130^Cas^, regulates focal adhesions and assembly of the cell–matrix adhesions [455,456,457,458].	Polyuria, polydipsia, secondary enuresis, growth retardation, anemia (*n* = 40) [417]. Juvenile NPH [451] [OMIM #256100, *607100]	--
*NPHP2/INVS* Nephrocystin-2/inversin	Localizes to primary cilia of renal tubular cells [459]. Co-IP ^1^ with nephrocystin-1 [459].	Enlarged kidneys, possible antenatal oligohydramnios and situs inversus, ADPKD-like features, cardiac abnormalities, ESRD ≤ 5 yo ^3^ [435]; [OMIM #602088, *243305]	*Inversin (Invs)*
*NPHP3* Nephrocystin-3	Ciliary structural protein functions in convergent extension at the embryonic Kupffer’s vesicle [460,461], genetic interaction with *Nphp2*/*invs* (zebrafish) [460].	Morpholino-mediated knockdown of zebrafish *nphp3* caused pronephric tubule cysts, inverted heart jogging (22.5%), heterotaxia (~7%), hydrocephalus, and situs inversus (*n* = 322) [460]. Adolescent and Infant NPH, liver fibrosis, RP ^2^, Meckel–Gruber syndrome. Ductal plate malformations, enlarged multicystic dysplastic kidneys, oligohydramnios, liver cysts, biliary cirrhosis, cystic pancreas, aortic stenosis, atrial septal defect [461], hepatic fibrosis, tapeto-retinal degeneration [462]. [OMIM #604387, *608002]	*nphp3*
*NPHP4* Nephrocystin-4	Interacts with nephrocystin-1 and 8 and α-tubulin [436,437]. Nephrocystins-4 and -1 interact with PALS1/PATJ and Par 6 (epithelial morphogenesis) [463].	Renal cysts, atrial and/or ventricular septal defects, pulmonary atresia [464]. RP ^2^, color blindness, Usher syndrome (hearing loss + RP ^2^), developmental delay [465] [OMIM #606966, *607215]	*nphp4*
*NPHP5/IQCB1* Nephrocystin-5	Localized to the primary cilia in renal tubular epithelial and retinal cells. Co-IP ^1^ with nephrocystin-5 and RPGR [463].	RP ^2^, kidney disease, Senior–Løken syndrome. [OMIM *609327]	*CG10649*
*NPHP6/CEP290* Nephrocystin-6	Interacts with Rab8a (ciliary biogenesis) and CP110 (inhibiting primary cilia formation) GTPases [466].	Joubert Syndrome, tapetoretinal degeneration, bilateral congenital amaurosis, nystagmus, retinal coloboma, cerebellar vermis aplasia/hypoplasia, ESRD, ataxia, intellectual disability, occipital meningoencephalocele [439]. [OMIM *610142]	*Cep290*
*NPHP7/GLIS2* Nephrocystin-7, Gli-similar protein	Repressor of Wnt/β-catenin/TCF-induced gene expression, transcriptional repressor [467], nuclear protein [468], normal kidney function, pancreatic development.	Renal failure before 8 yo ^3^ [OMIM #611498, *608539]	*Sugarbabe* (*sug*)
*NPHP8/RPGRIP1L* Nephrocystin-8/retinitis pigmentosa GTPase regulator interacting protein 1-like	Regulates murine Nphp4 and Nphp6/Cep290 (vertebrate transition zone assembly) [469].	Partial growth deficiency, polydactyly, scoliosis, pituitary agenesis. [OMIM *RPGRIP1L]	--
*NPHP9/NEK8* Nephrocystin-9/Never in mitosis A-related kinase 8 protein	Interacts with cyclin A-CDK, protects replication fork dynamics, reduces DNA damage in S phase and replication stress, coordinates firing of replication origin and replication fork speed [431]. *NPHP9* knockdown arrested breast cancer cell proliferation, and inhibited migration and invasion [470,471].	One patient had infantile NPH, 1 patient had RP ^2^. [OMIM #613824, *609799]	*niki*
*NPHP11/TMEM67/MSK3* Nephrocystin-11/Meckelin	Ciliogenesis [444]; predicted similarity to Frizzled ligands [444,472,473,474].	Liver disease, Joubert Syndrome, Meckel Syndrome. [OMIM #613550, *609884]	*CG15923*
*NPHP1L/XPNPEP3* Nephrocystin-1-like/X-prolyl aminopeptidase 3	Localized to mitochondria (IMCD3 cells) [427]. N-terminal endopeptidase cleaving Met-Pro sequences [475].	Hypertension, cardiomyopathy, renal failure, seizures. [OMIM *613553]	*CG32454*
*NPHP16/ANKS6* *Nephronophthisis 16*	Localized to the ciliary inversin compartment and requires NEK8 for localization. Kinase–bound ANKS6 also activates NEK8 [476].	Echogenic kidneys, congenital heart defects, heart and intestines situs inversus, hepatic fibrosis, hypertension [477].	--

^1^ CO-IP (co-immunoprecipitation), ^2^ RP (retinitis pigmentosa), ^3^ yo (years old), OMIM# (disease phenotype), OMIM* (specific gene entry).

### 4.2. Factors Implicated in NPH

Below and in Table 9 are the NPH-linked factors.

#### 4.2.1. NPHP1, NPHP2/INV, NPHP3, NPHP4

The *NPHP1* gene encodes nephrocystin-1, which is expressed primarily in the collecting duct cells and localizes at the adherens junctions and focal adhesions of the tubular renal epithelial cells and the cilium [478]. At these locations, NPHP1 interacts with NPHP2/INV (inversin), NPHP3 and NPHP4 [459,461,479]. NPHP2/INVS interacts with β-tubulin and anchors NPHP3 and NPHP9/Nek8 to the cilia [426,459,461,479]. NPHP2/INVS and NPHP4 localize at the centrosome, antagonize canonical Wnt signaling at/near the basal body in PCP signaling, and interact with E-cadherin and catenins during normal kidney development [480,481,482]. NPHP3, NPHP1 and NPHP2/INVS also work in the Wnt pathway [461]. Mutations in human *NPHP3* result in adolescent and infantile NPH, liver fibrosis, and Meckel–Gruber Syndrome [413]. Similarly, a mouse *Nphp3* knockout model phenocopied human Meckel–Gruber syndrome, displaying situs inversus, congenital heart defects and embryonic lethality [461]. The NPHP4 protein is localized to primary cilia, the basal bodies and centrosome [483] and interacts with NPHP1, NPHP8, and α-tubulin [436,437]. NPHP4 and NPHP1 interact with epithelial morphogens PALS1/PATJ and Par6 [463]. Follow-up on the observation that *NEK8* and *ANKYRIN6* (*ANKS6*) mutations yielded similar phenotypes revealed that eight NPH patients were bearing *ANKS6* mutations and that the NEK8 and ANKS6 proteins co-immunoprecipitated [484]. ANKS6 contains a C-terminal sterile alpha motif (SAM, typically a protein–protein interaction module) [484]. Consistently, in mice, Anks6, Anks3 and Bicc1 formed a complex and recruited several Nphp proteins via the ankyrins [485]. Anks3 and Anks6 both bound Bicc1; Anks3 inhibited the Bicc1 homopolymerization, while Ank6 recruitment restored it, presumably affecting Bicc1-dependent post-transcriptional regulation [486].

#### 4.2.2. NPHP5/IQCBI, NPHP6, and NPHP8/RPGRIP1L

Consistent with the presentation of retinitis pigmentosa in patients with *NPHP5* and *NPHP6* mutations, the NPHP5/IQCB1 and NPHP6 proteins were found to be associated in the primary cilia of both MDCK renal epithelial cells and photoreceptors [419,487]. Mutations in *NPHP6/CEP290/nephrocystin-6* cause Joubert syndrome, which is associated with NPH [439,440]. NPHP6/CEP290 localizes at the centrosome and mitotic spindle and interacts with activating transcription factor 4 (ATF4), which is known to contribute to renal cystogenesis [439]. Pathogenic variants of *NPHP6/CEP290* induce DNA replication stress and elevated cyclin-dependent kinase (CDK) [431,433]. These functions appear conserved in vertebrates, as a zebrafish *Nphp6* knockdown phenocopied NPH by displaying renal cysts, retinal degeneration and cerebellar malformation like human Joubert Syndrome [439]. The NPHP8/RPGRIP1L, or retinitis pigmentosa GTPase regulator interacting protein 1-like (RPGRIPL1) protein, colocalizes with NPHP4 and NPHP6 at both centrosomes and basal bodies [437]. Typically, NPHP8/RPGRIP1L interacts with NPHP4, which is functionally important because missense mutations reducing the RPGRIP1L-NPHP4 interactions are pathogenic and combine with partial growth deficiency, polydactyly, scoliosis, and pituitary agenesis [413,442]. In mouse IMCD3 cells, NPHP8/RPGRIP1L localized to the cilium and basal body and to the connecting cilium of photoreceptors [488]. In rat kidneys, NPHP6/CEP290, RPGRIP1L, and NPHP4 were found at the basal bodies of cilia in the renal tubular cells. Accordingly, in a 68-family cohort of individuals affected by Joubert syndrome, three individuals, or 5%, harbored *RPGRIP1L* mutations and exhibited variable phenotypes [442]. Unlike RPGRIP1 mutations, RPGRIP1L mutations are associated with renal but not retinal defects, possibly because of partial functional redundancy [442], with the caveat that some of the defects may not have been visible yet because of the young age of these individuals.

#### 4.2.3. NPHP7/GLIS2 and NPHP9/NEK8

*NPHP7* encodes Gli-similar protein 2 (GLIS2), which is a Kruppel-like zinc-finger transcription factor isolated in a large Cree Indian kindred found at the primary cilia and nucleus [489]. NPHP7/GLIS2 is found both in the nucleus and at the base of the cilium, affects epithelial–mesenchymal transition and fibrosis, and may mediate the consequences of *PKD1* dysfunction [255,489]. NPHP9/NEK8 (never in mitosis A-related kinase 8) is found at the centrosome in dividing cells and at the base of the cilium in ciliated cells [470,490]. In renal cells, NPHP9/NEK8 regulates the Hippo signaling factor YAP [175]. Cultured patient fibroblasts and murine inner medullary collecting duct (mIMCD3) cells bearing *Nek8* mutations revealed that NPHP9/NEK8 functions in ciliogenesis, proliferation, apoptosis, DNA damage response, and epithelial morphogenesis [175,431,433]. *NPHP7/GLIS2* mutations result in, hyper-phosphorylation of the H2A histone family member X (H2AX) and increased DNA breaks in the adolescent or adult kidney as well as the mouse *Glis2* model kidney [491]. In renal cells, NPHP9/NEK8 and NPHP4 deliver the unphosphorylated form of the Hippo signaling factor TAZ to the nucleus [492].

#### 4.2.4. NPHP1L/XPNPEP3 (NPH-like Nephropathy 1)

A conserved cytosolic (Human Protein Atlas) and mitochondrial [493] protein, NPHP1L/XPNPEP3 co-immunoprecipitated with components of the mitochondrial respiratory chain complex I, stabilized the complex, and may promote mitochondrial protein maturation [493]. *Xpnpep3* knockout mice displayed stress-induced ciliary elongation, renal tubule dilation, and fibrosis [493].

#### 4.2.5. NPHP11/MKS3/TMEM67 (Meckelin)

Meckelin is found in the primary cilia and plasma membrane. *NPHP11* mutations are present in NPH-like ciliopathies with liver disease, Joubert Syndrome and Meckel–Gruber Syndrome [444].

#### 4.2.6. Myc, cAMP, mTOR

Similar to ADPKD and ARPKD, Myc, cAMP and mTOR pathways are also dysregulated in NPH, favoring cell growth and proliferation (Figure 2) [494,495,496].

#### 4.2.7. Wnt, Hippo, Hedgehog

Both canonical and non-canonical Wnt pathways are affected in NPH. In a mouse model, Nphp2/Invs acts as the regulatory switch between the two to promote both Wnt pathway downregulation by targeting Dishevelled for degradation in the cytoplasm and convergent extension in development [481]. *Nphp3* loss-of-function affects oriented cell division [461]. Early evidence of abnormal activation of the Hippo pathway and its interaction with the Wnt cascades was confirmed recently [172,415,492,497,498,499]. In several *Nphp1* knockout models, the protein Kibra abnormally activated the Hippo pathway, causing fibrosis [500]. In human *NPHP1*^−/−^ kidney organoids, disrupted binding of NPHP1 to LATS1/2, a critical Hippo pathway component, promoted fibrosis through interleukin-1β signaling [501]. These changes were reversed by treatment with HIPPO inhibitor verteporfin [501]. Similarly, Hedgehog was also found to contribute to NPH. Primary collecting ducts explanted from *Nphp6* mutant mice and cells from a patient with Joubert Syndrome, and NPH revealed normal Wnt/WNT signaling with Hedgehog dysregulation that could be rescued by Hedgehog agonist purmorphamine [502]. Proteomics identified three NPHP complexes: MKS, involved in Hedgehog signaling, apical NPHP-1-4-8 (especially important for renal disease), and NPHP5-6 at the centrosome [503]. Therefore, the Wnt, Hippo and Hedgehog pathways are all dysregulated in NPH and are potential therapeutic targets [504].

**Table 9:** Factors contributing to NPH.

Molecular Trait	Increased/Activated/GOF or Decreased/Inactivated/LOF	Functions	Drosophila Ortholog
Myc	GOF ^1^ [495]	Pro-proliferation	*d-Myc*
mTOR	GOF ^1^ in the cyst-lining epithelium of NPH models LPK ^2^ rats [494] and zebrafish *msk1* morphants [505]	Nutrient sensing, cell growth and proliferation, autophagy [207]	*mTor*
cAMP	GOF ^1^ [495]	Second messenger and signal transduction	cAMP (molecule)
PCP, canonical	LOF ^3^, Defective. NPHP3 LOF ^2^ produces typical PCP defects [461]	NPHP2/INVS regulates canonical and non-canonical Wnt signaling [481]	wingless (wg) pathway
PCP, Non-canonical Wnt signaling	LOF ^3^, Defective. Mutations in *DCDC2* (NPHP type 19) lead to loss of cilia [506]	NPHP2/INVS regulates canonical and non-canonical Wnt signaling [481]	PCP pathway (*frizzled*, *fz*; *disheveled*, *dsh*)
Hippo	LOF ^3^, Defective Hippo pathway in *NEK8* mutants [175]	Cell proliferation, survival, organ size. Contributes to severe renal cystic syndromes [175]	Hippo Pathway
Hedgehog	LOF ^3^, Yes, kidneys from *Cep290^LacZ/LacZ^* mice with hedgehog LOF ^2^ had increased Gli3 repressor [502]	Signaling pathway activated by primary cilia and regulates cell fate [210]	*Hedgehog (hh)*
DNA Damage Repair (DDR) pathway	GOF ^1^	Increased sensitivity to DNA damage in NPHP factors CEP164 or ZNF423 knockdowns [430]	--
PALS1/PATJ	LOF ^3^ [463]	Interacts with NPHP1 and NPHP4	*stardust (sdt)*
Apoptosis	GOF ^1^ [507]	Increased apoptosis	Apoptosis pathways (e.g., *Reaper*, *Hid*, *Grim*)
Interstitial Fibrosis	GOF ^1^, both renal and extrarenal, ESRD ^4^ driver (e.g., liver, [415]).	Pro-fibrotic, NPH-like elevated Sox9 and Wnt4 in *Fbxw7* kidney knockouts [508]	Unknown
BicC	--	Possible involvement through interaction with ANKS3 and ANKS6 [509]	*Bicaudal C (BicC)*

^1^ GOF (gain-of-function), ^2^ LPK (Lewis polycystic kidney disease), ^3^ LOF (loss-of-function), ^4^ ESRD (end-stage renal disease).

### 4.3. Metabolic Signature of NPH

Comparatively less studied than ADPKD and ARPKD, metabolic changes are likely to occur in NPH. Suggestive evidence includes that gene knockouts of *NPHP16/ANKS6* and NPH-related *ANKS3* (encoding ankyrin 3) reduced metabolism and proliferation of the murine IMCD3 cells [510,511]. Note that ANKS6 and ANKS3, NEK8, INVS, and NPHP3 proteins (Table 8) all interact with each other and mitochondrial proteins [484], and NPHP1L/XPNP3 is a mitochondrial protein (Table 8), increasing the likelihood of their involvement in the cellular energy pathways [493]. Renal fibrosis, prominent in NPH, has also been linked with metabolic disruptions and mitochondrial dysfunction [512,513,514,515].

### 4.4. Outlook on Factors Implicated in NPH

A polycystic kidney disease, NPH also displays distinctive phenotypes from ADPKD and ARPKD, including prominent fibrosis and retinal involvement (Figure 1, Table 7A,B). Possibly forming a shared core molecular foundation, several NPH proteins are found at the cell junctions and the cilia, several of the pathways disrupted by ADPKD and ARPKD and some of the protein factors implicated (e.g., c-Myc) and interactors (e.g., murine Bicc1) are also relevant for NPH (Table 9, Figure 2). However, their precise roles in each pathology are incompletely defined. While the polycystins and fibrocystin are clearly involved in extracellular vesicles as both cargo and functional factors, there is no evidence of similar nephrocystin functions. A recent report on a type of *C. elegans* male sensory neurons showed that a polycystin 2 homolog and a NPHP-4 homolog at the transition zone were needed to sustain extracellular vesicle-mediated signaling [516]. Whether this observation can be translated to similar renal functions remains to be defined.

### 4.5. NPH Treatment

As for other ciliopathies, there are currently only supportive therapeutic options for individuals with NPH (Table 10). Efforts are directed toward managing anemia, secondary hyperparathyroidism, metabolic bone disease and blood pressure [517]. Eventually, kidney transplantation is considered [415]. Several pharmacological studies in animal models are ongoing; however, no molecules have advanced to clinical trials [415], and ongoing clinical trials focus on the treatment of stones (Table A3). The ADPKD drug tolvaptan was tested on one 82-day-old patient and did not significantly delay the functional decline of the kidney [518]. Like ADPKD, the mTOR inhibitor rapamycin was also tested on the *Pcy* murine Nphp3 model, where it was found that rapamycin slightly slowed down the deterioration of renal function and histopathological progression in late stages of NPH [519]. Roscovitine, a cyclin-dependent kinase inhibitor, and analog S-CR8 were used to downregulate this kinase in renal cells from a Joubert Syndrome patient [520].

## 5. Renal Cystic Disease, Development, and Drosophila Models

### 5.1. A Developmental View of Renal Cystic Disease

ADPKD, ARPKD, and NPH appear as distinct phenotypes that are part of a continuum (Figure 2). Severe ADPKD phenocopies ARPKD [64]; NPH can phenocopy ARPKD [331,528] and, more rarely, ADPKD [5,64]. Next-generation sequencing confirmed genetic heterogeneity in individuals presenting with renal cystic dysplasia [529]. As examined above and summarized in Figure 3, ADPKD, ARPKD and NPH appear to implicate numerous common factors and pathways, many of which have developmental functions. In both embryonic patterning and organogenesis, PCP provides positional information to individual cells and epithelial sheets during development in response to morphogenic gradients (e.g., Wnt/Frizzled signaling) and mechanical forces through mechanisms to be precisely defined [530,531,532,533,534,535]. During renal development, canonical Wnt signaling regulates cell division and non-canonical Wnt signaling regulates oriented cell division through PCP; when non-canonical Wnt signaling is defective, canonical Wnt signaling disrupts apical–basolateral polarity within the renal epithelial cells, which promotes cyst formation [413]. The developing nephron responds to the regional expression of several Wnt family members [164]. Ureteric bud development and branching morphogenesis also require PCP [535,536,537]. Relevant for cystic pathology, kidney lysates from ARPKD patients contained higher mRNA levels for PCP factors *WNT5*, *VANGL2*, *ATMIN* and *SCRIB* [392]. However, the presence of several paralogous genes for PCP factors complicates studies to define their precise contribution to renal tubule and kidney development [538]. Growth factor signaling and regulators of cell proliferation and remodeling (e.g., MYC, mTOR, SRC, TNF-α, EGF) are both important in development and relevant for renal cyst formation and growth [87,539]. The variable severity of the cystic phenotype has informed a view of renal cystic disease as a temporal continuum from early sensitization (likely from defective canonical and non-canonical Wnt signaling) to early cyst formation and later growth [394,540]. ADPKD is typically regarded as an adult-onset malady and often diagnosed once kidney damage has compromised renal function; however, newborns already have small cysts [2] and are presumably already sensitized to cyst emergence. Understanding these early mechanisms and the developmental arc of PKD would be beneficial to devise early management plans to delay functional damage [394]. Early pro-cystic changes are likely to affect several cellular pathways, and over time the cellular network adjusts as cell and tissue damage continue and fibrosis sets in. Network-level disruption of cell signaling and metabolism, heterogeneous presentation with individual severity, and degenerative trajectory all suggest that variable involvement of shared factors, the type of mutations, and the genetic variants affecting the activity of the interacting partners in macromolecular complexes all contribute to the observed phenotypes. Thus, genome-wide investigation will likely be needed to unravel the workings of the underlying genetic network. Human genome-wide association studies (GWAS) correlate genomic variants with pathological traits and may reveal associations, provided a large enough population of affected individuals is available. In contrast, GWAS in *Drosophila* can have complete genomic coverage, and the large collection of genetic tools enables rapid follow-up causative studies [541,542].

### 5.2. Modeling PKD in Drosophila

**Figure 4:**
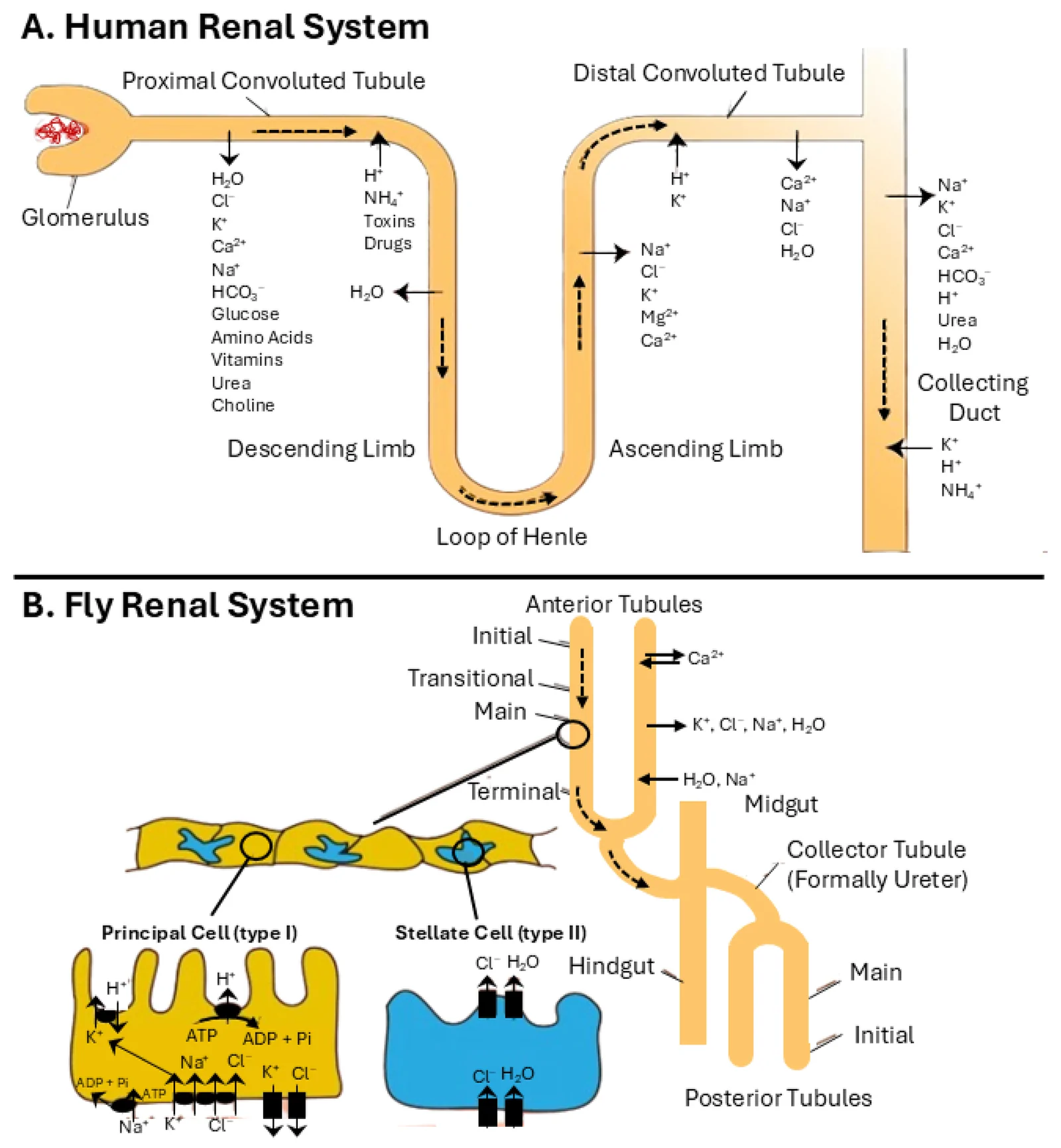
**Conservation of the human and fly renal systems.** Functionally different regions with ion and molecule transport (arrows) indicated. (**A**) Schematic human glomerular renal tubule (nephron). (**B**) Drosophila Malpighian tubule pairs (anterior, posterior, right). Malpighian tubules are formed by two major cell types, principal (gold), and stellate (blue) cells with distinct functions and properties (shown). Modified from [549].

The limitations of therapeutic options for treating PKD emphasize the urgent need to better understand the pathological mechanisms of the renal cysts. However, substantial phenotypic variability and numerous genetic contributors and modifiers challenge mechanistic studies based on single-gene approaches. On the other hand, genome-wide approaches designed to capture the genetic networks perturbed by the renal cysts may provide new, broader perspectives and help untangle the observed phenotypic complexity. The presence of several homo- and paralogous genes for key factors implicated in renal cystic disease complicates the study in vertebrates. In contrast, Drosophila has been extensively used in developmental, mechanistic, and genetic studies to dissect complex biological problems [543]. Low genetic redundancy overcomes the difficulty of testing specific questions in cell culture or mammalian models [544]. Despite obvious anatomical differences, conservation of ~75% of the genes involved in human disease and their pathways, a wealth of genetic tools, mutant and transgenic lines, and an exceptionally collaborative research community enable the iterations needed in mechanistic studies and have yielded translationally valuable knowledge [545,546,547,548]. In Drosophila, the Malpighian tubules are functionally equivalent to the tubular portion of the human nephron that is the site of PKD-like renal cyst formation (Figure 4) [549,550]. Unlike the vertebrate nephron, the fly Malpighian tubules can be precisely micro-dissected with preserved functionality suitable for physiological [551,552] and molecular assays. The Malpighian tubules consist of three specialized regions: proximal, secreting fluids into the tubules; intermediate, secreting water and potassium chloride; and terminal, reabsorbing sodium and potentially water (Figure 4) [549]. Transcriptomics studies of the renal tubule have suggested that comparatively, the renal function may be exceptionally well conserved due to the constraints of nitrogen excretion [352,549,553]. Malpighian tubule development in Drosophila and vertebrates also follows similar principles [549]. Thus, with proper perspective and careful use of the fly genetic tools, Drosophila may offer a unique opportunity to rapidly decipher the core components of complex pathologies and accelerate lengthier vertebrate studies [554]. Although flies do not have ciliated epithelia, the Drosophila genome contains homologs for most ciliary proteins and was instrumental in the discovery of relevant ciliary pathways and conserved functions [555,556,557,558,559]. An excellent example of valuable translational knowledge, the fly PC2 homolog, Almost There (Amo), is required for sperm motility and fertilization [104,560]. A forward screen in Drosophila identified the mitochondrial SLC25A25 transporter as a conserved downstream effector of PC2 at the vertebrate renal cilium [287]. Downstream of the human *PKD1* and murine *Pkd1* genes [128], *BicC/BICC1/Bicc1* is also implicated in renal cyst pathology. An interactor of PC1 and PC2 in human cells [204] and one component of the Nphp complexes [485] in the mouse, BICC1/Bicc1 protein is a bona fide renal regulator, and its mutations cause cystic phenotypes from flies to humans [128,170,199,200,201]. Drosophila *BicC* mutants exhibit cystic Malpighian tubules with several PKD-like features, including progressive tubular degeneration, similar regional cyst distribution and compromised renal function (Table 11) [128]. Molecularly, the Malpighian tubules from two *BicC* allelic combinations displayed PKD-like causative Myc over-expression, mTOR hyperactivity [128] and conserved pharmacological response to rapamycin [128] and mimics of the second mitochondria-derived activator of caspases (Smac) [158]. Smac mimics selectively sensitize neoplastic cystic cells fueled by TNF-α to apoptosis. One Smac mimic exhibited mild cyst-reducing activity in a murine PKD model [157]. Suggestive of evolutionary conservation of cystic-relevant pathways, three distinct Smac mimics ameliorated the cystic *BicC* tubules in flies and exhibited differential regional efficacy [158]. The *BicC* renal phenotype and its amelioration following melatonin treatment [561] also implied the dysregulation of other PKD-relevant pathways to be defined because melatonin reduces cancer cell proliferation by simultaneously regulating several growth factor pathways, e.g., TOR, MAPK, EGF [92]. Thus, fly *BicC* mutants may provide knowledge potentially translatable to PKD and NPH. Similarly, a fly model of renal fibrosis recapitulates steroid-induced excessive deposition of collagen-like pericardin that damages the nephrocytes (analogous to the vertebrate glomerular podocytes) yielding proteinuria [562] and may give insight into renal fibrosis that was previously thought to be absent in Drosophila. Several of the factors contributing to ADPKD, ARPKD and NPH have fly homologs, often with known developmental roles (Table 2, Table 5 and Table 9). Between 80 and 90% of the enzymes in core metabolic pathways relevant to PKD (e.g., glycolysis, TCA, insulin) and pathway topology of the regulatory networks (e.g., insulin, mTOR, AMPK) are conserved between Drosophila and humans [563,564,565,566], opening the possibility of strategic use of Drosophila to unravel the intricacies of renal cyst pathology. Importantly, renal cystic disease appears to entail layered defects in the function of developmental factors governing cell polarity, division, and metabolism shared in part among ADPKD, ARPKD, and NPH. Their characteristic variability in phenotypic presentation, severity, and disease progression points to the existence of numerous modifiers and networks to be defined, ideally through genome-wide approaches. Drosophila, with its wealth of genetic resources and streamlined anatomy and lean yet refined genetic structure could prove uniquely effective in mapping the renal cystic networks.

## 6. Conclusions

The renal cystic diseases ADPKD, ARPKD and NPH all feature high variability in phenotypic severity and speed of progression towards ESRD due in part to their polygenic nature. Numerous factors, both shared and distinctive, are expected to contribute variably to the resulting renal cystic phenotypes. Also typical, intra-familial variability strongly suggests the existence of yet-to-be-defined genetic modifiers, a possibility supported by emerging transcriptomics and metabolomics data. These studies have also indicated metabolic remodeling that implies network-level disruptions in the cystic renal tubules. However, the presence of paralogs for known causative genes that may encode proteins with at least partially overlapping functions complicates the functional studies of ADPKD in mammals and other vertebrates. For example, *PKD1* paralogs *PKD1-like* (*L*) *1, PKD1L2*, and *PKD1L3* encode proteins that can form complexes with PC2 and PC2-like proteins at the cilium, and the human genome contains 19 *WNT* genes clustering in subclasses [576,577,578,579,580]. The staggeringly long list of factors variably contributing to these polycystic kidney diseases and particularly to the best-studied ADPKD suggests that the functional relationships among these factors, or their network properties, may be as important as their identity. However, these network properties remain to be defined. More research is needed to decipher the formidable complexity of ADPKD, ARPKD and NPH and their shared and unique contributors, sensitizing components and environmental influences for better prognosis and treatments. Because of the developmental character of the renal cystic diseases, the strategic deployment of rodent, zebrafish, and invertebrate developmental models to address unresolved aspects may provide an integrated view of the conserved core networks and the refined layers of duplication and differentiation typical of human renal function.

## Figures and Tables

**Table 3:** ADPKD treatments.

Treatment	Used To	Long Term Efficacy
Sodium-restrictive diet	Decrease kidney growth and eGFR decline [309].	Yes
Fluid intake	Increase urine volume and decrease urine osmolality.	Yes
Hemodialysis	Remove metabolic waste and excess fluids.	Yes
Peritoneal dialysis	Remove metabolic waste and excess fluids.	Yes
Kidney transplant	Restore kidney function by explanting cystic kidneys or adding one extra kidney. Lifesaving.	8–20 years [310]
Tolvaptan *Repurposed V2R antagonist*	Slow kidney functional decline (eGFR). Reduce pain (*n* = 1445), ages 18–50 with TKV ^1^ ≥ 750 mL, estimated creatinine clearance of ≥ 60 mL/minute [292].	Potential hepatotoxicity, unknown long-term efficacy [311]
RGLS4326 *Short oligonucleotide inhibitor of miR-17* (Anti-*miR-17* oligonucleotide)	Subcutaneous injection, *miR-17* target de-repression (e.g., *Pkd1*, *Pkd2* mRNAs). *Pkd2*-KO ^2^ mice displayed reduced kidney-to-body weight ratio and epithelial cystic cell proliferation [312].	--
RGLS8429 *Farabursen* Next-generation successor of RGLS4326	*miR-17* target de-repression (e.g., *Pkd1*, *Pkd2*). Lower cyst volume (mouse and human ADPKD cell lines). In murine collecting duct cells, cyst prevention (early treatment) or stabilization (late treatment) [313].	--
ACE inhibitors and ARBs	Manage high blood pressure.	Unknown

^1^ TKV (total kidney volume), ^2^ KO (knockout).

**Table 4:** (**A**) Renal-associated manifestations and incidence of ARPKD, ranked by incidence. (**B**) Extrarenal manifestations and incidence of ARPKD, ranked by incidence.

Symptom	Frequency
(**A, renal**)	
Cysts	100%
ESKD	50% (*n* = n/a ^1^) ≥ 20 yo ^2^ [38]
Urinary tract infection	~30% (*n* = 115)
Metabolic acidosis	15% of CKD patients (*n* = 160) [335]
GFR decline	~5.01% (*n* = 16 children ≤ 10 yo ^2^) ~11.5% (*n* = 6 ≥ 10 yo ^2^) [336]
Mild proteinuria	n/a ^1^
Recurrent urinary tract Infections	n/a ^1^
(**B, extrarenal**)	
Arterial hypertension	80% of children (*n* = 186) [337]
Systemic hypertension	55–75% (*n* = 115) [338,339], ~75% (*n* = 164) [325]
Biliary abnormalities	70% (*n* = 72) [340,341]
Pulmonary hypoplasia	30–40% (*n* = n/a ^1^) [337]
Oligohydramnios	30–40% mortality (*n* = 209) [341]; 33% oligohydramnios (*n* = 6) [342]
Respiratory distress (peri-/neonatal)	30–50% (*n* = n/a ^1^) [337], 13% (*n* = 52) [343]
Liver disease	~46% (*n* = 115) [338] 26% (*n* = 73) [339,340]
Ductal plate malformation/congenital hepatic fibrosis	n/a ^1^ [337]; 25% (*n* = 20) [344]
Hyponatremia	15% before 1990 (*n* = 40) [319]; 26.5% after 1990 (*n* = 151) [319]; 79% (*n* = 55) [345]
Acute bacterial cholangitis	9.4% (*n* = 32) [341,346]
Intra- and extracranial aneurysms	(*n* = 1), single case report [337]
Pancreatic cysts and/or fibrosis	n/a ^1^
Club feet	n/a ^1^

^1^ n/a (not available), ^2^ yo (years old).

**Table 6:** ARPKD treatments.

Treatment	Used To
Peritoneal dialysis, hemodialysis	Remove excess waste and fluids. ESRD ^1^ [411]
Unilateral or bilateral nephrectomy	Alleviate diaphragmatic constriction and feeding intolerances from the enlarged kidneys [411]
Kidney transplant	Restore kidney function, lifesaving [411]
Artificial ventilation	Regulate breathing [411]
Blood pressure medication/antihypertensive drugs, salt restriction	Control hypertension [411]
Nasogastric or gastrostomy tube	Supplemental feeding counters food intolerance and stunted growth [328]

^1^ ESRD (end-stage renal disease).

**Table 10:** NPH treatments.

Treatments	Used To
Correction of water and electrolyte imbalances [521]	Correct polyuria, polydipsia
Phosphate binders, vitamin D, calcimimetics [522]	Correct secondary hyperparathyroidism
Recombinant erythropoietin [523]	Counter anemia
Blood pressure medication [524]	Normalize hypertension
Growth hormone treatment [525]	Counter growth retardation
Dialysis or kidney transplantation [526]	Reduce corticomedullary cysts
Dialysis or kidney transplantation [527]	Counter ESKD ^1^/ESRD ^2^

^1^ ESKD (end-stage kidney disease), ^2^ ESRD (end-stage renal disease).

**Table 11:** Phenotypes of Drosophila *BicC* mutants relevant to human cystic kidney disease.

Phenotype	*Drosophila BicC* Mutants	Human ADPKD	Human ARPKD	Human NPH
Cysts	Yes [128]	Yes [1,2,3,337]	Yes [1,2,3,337]	Yes [10,415]
Renal fibrosis	Unknown	Yes [1,2,3,567]	Yes [568]	Yes [10,415,447]
Impaired renal function	Yes, salt stress sensitivity [128]	Yes, impaired GFR [1,2,3]	Yes [2]	Yes [10,415,447]
Infertility	Yes [569]	Yes [3,570]	n/a ^2^	n/a ^2^
Myc upregulation	Yes [128]	Yes [116]	Yes [375]	Indirect evidence [10,414,571]
MTOR activation	Yes [128]	Yes [572]	Yes [389]	Yes [573]
TNF-α upregulation	Yes, implied by Smac mimicry response [158,574]	Yes [157]	n/a ^2^	n/a ^2^
Wnt activation	Likely, in the ovary [171]	Yes [575]	Yes [575]	Yes [575]
Src upregulation	Likely, in the ovary [171]	Yes [208]	Yes [373]	n/a ^2^
BicC LOF ^1^	Yes ^2^	Yes, downregulation in *PKD1*, *Pkd1* LOF ^1^ [128,203]. Molecular interaction with PC1, PC2 [204]	n/a ^2^	Yes, interaction with ANKS3 and ANKS6 [509]

^1^ LOF (loss-of-function), ^2^ n/a (not available).

## Data Availability

All the data sources are cited or linked in the document.

## Abbreviations

The following abbreviations are used in this manuscript:

ADPKD	Autosomal dominant polycystic kidney disease
ARPKD	Autosomal recessive polycystic kidney disease
ATF4	Activating transcription factor 4
ADH	Anti-diuretic hormone, a.k.a. vasopressin
AVP	Arginine vasopressin
*BicC*	*Bicaudal C*, gene, Drosophila
BicC	Bicaudal C, protein, Drosophila
*BICC1*	*BICAUDAL C*, gene, human
BICC1	BICAUDAL C, protein, human
*Bicc1*	*Bicaudal C*, gene, murine
Bicc1	Bicaudal C, protein, murine
cAMP	3’-5’ cyclic adenosine monophosphate
CDCA	Cilia-dependent cyst activation signal
CDK	Cyclin-dependent kinases
DZIP1L	DAZ-interacting zinc finger protein 1 like
ESKD	End-stage kidney disease
ESRD	End-stage renal disease
GFR	Glomerular filtration rate
GOF	Gain-of-function
GPCR	G-protein coupled receptor
GUR	Genetically unresolved
HEK 293T	Human embryonic kidney, cells
IFT	Intraflagellar transport system
LOF	Loss-of-function
MAPKK	Mitogen-activated protein kinase kinase
MDCK	Madin–Darby canine kidney, cells
MEK	Mouse embryonic kidney, cells
NPH	Nephronophthisis
Orpk	Oak Ridge polycystic kidney
PC	Polycystin, human
Pc	Polycystin, murine
PCP	Planar cell polarity
*PKHD1*	*POLYCYSTIC KIDNEY AND HEPATIC DISEASE* 1, gene, human
*Pkhd1*	*Polycystic kidney and hepatic disease 1*, gene, murine
PLD	Polycystic liver disease
PPAR	Peroxisome Proliferator-Activated Receptors
PKD	Polycystic kidney disease
RCC	Renal cell carcinoma
RHEB	Ras homolog enriched in brain
RP	Retinitis pigmentosa
RPGRIP1L	Retinitis pigmentosa GTPase regulator interacting protein 1 like
RRT	Renal replacement therapy
RTK	Receptor tyrosine kinases
Smac	Second mitochondria-derived activator of caspases
TGF-β	Transforming growth factor beta
TNF-α	Tumor necrosis factor alpha
TRP	Transient receptor potential
TSC	Tuberous sclerosis complex
UTI	Urinary tract infection
UTR	Untranslated region
ZO-1	Zonula Occludens-1

## Appendix A

Table A1, Table A2 and Table A3 (see text).

**Table A1:** ADPKD clinical trials (from Clinicaltrials.gov [581]).

Clinical Trial	Testing	Location/Sponsor	Current State
NCT06391450	Study of Empagliflozin	Hanover Medical School, Germany	Phase 4
NCT05510115	Feasibility of study of Empagliflozin	University of Colorado, Denver	Phase 2
NCT06496542	Renal oxygen consumption, insulin sensitivity and daily caloric restriction	University of Colorado, Denver	Phase Not Applicable
NCT07217158	Role of ROS ^1^ and cAMP ^2^-PKA ^3^ biomarkers	Mayo Clinic	Observational
NCT06747572	*PKD1* gene variant groups	Vertex Pharmaceuticals Incorporated	Observational
NCT05193981	Evaluate homocysteine metabolism and endothelial function	Mayo Clinic	Observational
NCT06289998	Tamibarotene	Japan	Phase 2
NCT07016282	Real-world data simulation of ADPKD trials	Mario Negri Institute for Pharmacological Research	Observational
NCT05288998	Intrarenal microvasculature	Mayo Clinic	Observational
NCT06688981	Artificial Intelligence-based image processing methods to advance the characterization of PKD	Mario Negri Institute for Pharmacological Research	Observational
NCT06036992	Study and management of cystic complications in ADPKD	University Hospital, Brest	Observational
NCT06193616	Outcome of ADPKD with octreotide LAR (Long-Acting Release)	Mario Negri Institute for Pharmacological Research	Observational
NCT07355114	Robotic vs. open nephrectomy for ADPKD	University of Bologna	Observational
NCT06594367	A possible founding *PKD2* mutation associated with variable phenotypes of ADPKD	Mario Negri Institute for Pharmacological Research	Observational
NCT03273413	Statin therapy in patients with early-stage ADPKD	University of Colorado, Denver	Phase 4
NCT05460169	Renal Denervation in ADPKD-RDN-ADPKD Study	University of Erlangen-Nürnberg Medical School	n/a ^4^
NCT04907799	Daily caloric restriction	University of Colorado, Denver	n/a ^4^
NCT06902558	Assess adverse events and Effectiveness of Intravenous Infusion of ABBV-CLS-628 in adult participants with ADPKD	AbbVie	Phase 2
NCT06435858	Short-term effects of an SGLT2 inhibitor on Divalent Ions in ADPKD	Cantonal Hospital Graubuenden	Phase 2
NCT07228364	Safety, Tolerability and Pharmacokinetics of AZD1613 in adults with ADPKD	AstraZeneca	Phase 1
NCT04344769	Characterization of the Nrf2 Response in patients with ADPKD	Mayo Clinic	Observational
NCT07280585	STOP-PKD: SGLT2-inhibition to improve prognosis in PKD	University of Cologne	Phase 3
NCT07260071	Hypertension in children and young people at risk of ADPKD	King’s College London	Observational
NCT04630613	NOX4 and related biomarkers in ADPKD	Mayo Clinic	Observational (Patient Registry)
NCT04939935	Implementation of Metformin therapy to ease decline of kidney function in PKD	The University of Queensland	Phase 3
NCT06800651	Trial of JMKX003142 in participants with rapidly progressive ADPKD	Jemincare	Phase 2

^1^ ROS (Reactive Oxygen Species), ^2^ cAMP (Cyclic AMP), ^3^ PKA (Protein Kinase A), ^4^ n/a (not available).

**Table A2:** Clinical trials for ARPKD (from Clinicaltrials.gov [582]).

Clinical Trial	Testing	Location/Sponsor	Current State
NCT04782258	Tests tolvaptan safety in infants and children with ARPKD (28 days to ≤18 yo ^1^).	Otsuka Pharmaceutical Development & Commercialization, Inc.	Phase 3
NCT07201025	Imaging Assessments of ARPKD Progression	The Cleveland Clinic	Observational
NCT06601829	Congenital hepatic fibrosis and ARPKD in Children	Sohag University	Observational
NCT01401998	ARPKD Database Study	Children’s Hospital of Philadelphia	Observational
NCT06065852	National Registry of Rare Kidney Diseases	UK Kidney Association	Observational (patient Registry)
NCT06147414	Development of Non-Invasive Prenatal Diagnosis for single-gene disorders	Assistance Publique—Hôpitaux de Paris	Observational

^1^ yo (years old).

**Table A3:** Clinical trials for NPH (from Clinicaltrials.gov [583]).

Clinical Trial Number	Testing	Location/Sponsors	Current State
NCT06922006	Feasibility of radiation-free percutaneous nephrolithotomy for kidney stones	University of California, San Diego	Observational Patient Registry
NCT04633811	Effect of weight loss on urinary oxalate excretion in obese calcium oxalate kidney stone formers	University of Alabama at Birmingham	n/a ^1^
NCT04051346	Dietary oxalate and innate immunity in kidney stone disease	University of Alabama at Birmingham	n/a ^1^
NCT02028559	Safety and effectiveness of the ultrasonic propulsion of kidney stones	University of Washington	n/a ^1^
NCT05353478	Reducing opioid prescription after kidney stone removal surgery	Mayo Clinic	Observational
NCT06615713	Evaluate the safety and effectiveness of steerable ureteroscopic renal evacuation (SURE) using the CVAC^®^ System	Calyxo, Inc.	n/a ^1^
NCT06635889	Intravesical bupivacaine on post-operative ureteroscopy pain	University of Chicago	Phase 2
NCT06974357	Evaluation of genetic abnormalities amongst calcium phosphate stone formers	Ryan L Steinberg	n/a ^1^
NCT06721975	Thulium fiber laser (TFL) vs thulio pulsed thulium:YAG (p-Tm:YAG)	University of California, San Diego	n/a ^1^

^1^ n/a (not available).
